# Dynamic Reconstruction of Yttrium Oxide‐Stabilized Cobalt‐Loaded Carbon‐Based Catalysts During Thermal Ammonia Decomposition

**DOI:** 10.1002/advs.202406659

**Published:** 2024-09-24

**Authors:** Yi Zhu, Hongfei Pan, Qi Li, Xiege Huang, Wei Xi, Haibo Tang, Wenmao Tu, Shihao Wang, Haolin Tang, Haining Zhang

**Affiliations:** ^1^ State Key Laboratory of Advanced Technology for Materials Synthesis and Processing Wuhan University of Technology Wuhan 430070 China; ^2^ R&D Center of Materials and Stack Technology for Fuel Cell National Energy Key Laboratory for New Hydrogen‐Ammonia Energy Technologies Foshan Xianhu Laboratory Foshan 528200 China; ^3^ Hubei Key Laboratory of Theory and Application of Advanced Materials Mechanics Wuhan University of Technology Wuhan 430070 China; ^4^ School of Chemical Engineering and Technology Tianjin University Tianjin 300382 China; ^5^ Hubei Key Laboratory of Fuel Cell Wuhan University of Technology Nr. 122 Luoshi Rd. Wuhan 430070 China

**Keywords:** ammonia decomposition, cobalt‐based catalysts, dynamic reconstruction, hydrogen production, yttrium oxide

## Abstract

Hydrogen production from the decomposition of ammonia is considered an effective approach for addressing challenges associated with hydrogen storage and transportation. However, their relatively high energy consumption and low efficiency hinder practical multi‐scenario applications. In this study, Y_2_O_3_‐stabilized catalysts with Co‐loaded onto porous nitrogen‐doped carbon (Y_2_O_3_–Co/NC) are synthesized by pyrolysis of Y(NO_3_)_3_‐modified ZIF‐67 under an inert atmosphere, followed by annealing in a reducing environment. The introduction of Y_2_O_3_ enhanced the recombination and desorption of N atoms and facilitated the gradual dehydrogenation of NH_x_ on the catalyst surface, resulting in improved catalytic activity for the thermal decomposition of ammonia. Benefitting from the electron‐donating properties of Y_2_O_3_ and N‐doped carbon, the optimized catalyst achieved a remarkable NH_3_ conversion efficiency of 92.3% at a high gas hourly space velocity of 20 000 cm^3^·$g_{\mathrm{cat}}^{-1}$·h^−1^ with an encouraging H_2_ production rate of 20.6 mmol·$g_{\mathrm{cat}}^{-1}$·min^−1^ at 550 °C. Moreover, the synthesized catalyst undergoes a fast‐dynamic reconstruction process, resulting in exceptionally stable catalytic activity during the thermal decomposition of ammonia, rendering it a promising candidate for carbon‐free energy thermocatalytic conversion technology.

## Graphical abstract

Y_2_O_3_‐Co/NC catalyst synthesized through the pyrolysis of Y(NO_3_)_3_‐modified ZIF‐67 exhibits exceptional thermal catalytic performance via small‐sized nanoparticles restricted by carbon support and rare‐earth oxide. Moreover, the synthesized Y_2_O_3_‐Co/NC catalyst undergoes a fast‐dynamic reconstruction process with a cyclic growth and splitting of Co nanoparticles during the ammonia thermal decomposition, suggesting a promising catalytic stability for practical application.

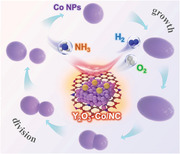

## 1 Introduction

The development and utilization of hydrogen energy—a green source of energy with high energy density of hydrogen (143 MJ·kg^−1^)—can effectively mitigate the energy crisis and environmental problems caused by the excessive exploitation of fossil fuels.^[^ ^1^, ^2^ ^]^ However, storage and transportation issues limit their widespread application because of difficulties in compressing hydrogen as a liquid. Owing to its high hydrogen content (17.6 wt.%) and large bulk energy density (11.5 MJ·L^−1^), hydrogen generation by ammonia decomposition is an efficient and feasible approach to address the aforementioned challenges, in addition to the direct usage of ammonia as fuels in internal combustion engines.^[^ ^3^, ^4^, ^5^, ^6^ ^]^ Nevertheless, the endothermicity of ammonia decomposition requires the reaction to operate at high temperatures, resulting in high energy consumption. Numerous studies have shown that the introduction of catalysts can significantly decrease the energy consumption by reducing the activation energy of the ammonia decomposition reaction.^[^ ^7^, ^8^ ^]^ For example, ruthenium‐based catalysts have been shown to demonstrate superior ammonia decomposition activity. However, their commercial application is difficult owing to the high‐cost and scarcity of ruthenium.^[^ ^9^, ^10^, ^11^ ^]^ Therefore, the development of effective ammonia decomposition catalysts with high economic benefits is essential for replacing Ru‐based catalysts.

Among non‐noble metal catalysts developed, cobalt‐based catalysts exhibit promising ammonia decomposition activity and potential for economic viability.^[^ ^12^ ^]^ For instance,^[^ ^13^ ^]^ Co nanoparticles embedded in a carbon matrix with an average particle size of 4.7 nm showed an ammonia conversion rate of 55% at 500 °C under a gas hourly space velocity (GHSV) of 15 000 cm^3^·$g_{\mathrm{cat}}^{-1}$·h^−1^. In another example,^[^ ^14^ ^]^ Co nanoparticles (≈30 nm in size) captured in carbon micropores, which were synthesized by a wet immersion method, exhibited a relatively high activity for ammonia decomposition (60%; GHSV = 5,200 cm^3^·$g_{\mathrm{cat}}^{-1}$·h^−1^) at 500 °C. Despite the notable examples, the widespread practical application of non‐noble metal catalysts in thermal reactions is scarce due to their susceptibility to deactivation at high temperatures and remarkably reduced activity at low temperatures.

Notably, incorporating metal nanoparticles with the appropriate support, such as carbon‐based materials, is an effective approach for achieving both high catalytic activity and stability owing to the improved dispersity of the metal species, and favorable electron transfer as a result of high conductivity and devisable porosity.^[^ ^15^, ^16^ ^]^ Carbon‐supported metal composites obtained by the pyrolysis of metal–organic framework (MOF) materials have attracted considerable attention because of their various desirable advantages, including a retained porous structure and uniformly dispersed metal particles, which improve the catalytic performance of ammonia decomposition catalysts.^[^ ^17^, ^18^, ^19^ ^]^ Specifically, a porous structure can facilitate the adsorption of ammonia by exposure of a greater proportion of catalytically active sites on the metal nanoparticles. ZIF‐67 is an MOF material assembled with cobalt ions as the central atom and 2‐methylimidazole as the organic ligand. Currently, Co‐based catalysts derived from ZIF‐67 show great potential for catalyzing ammonia decomposition for hydrogen production because of their high metal loading and dispersion. For example,^[^ ^20^ ^]^ Li et al. found that a nitrogen‐doped carbon‐coated Co catalyst produced from ZIF‐67 pyrolysis under a nitrogen atmosphere can effectively protect the aggregation of Co nanoparticles and achieve an ammonia decomposition rate of 80% at 500 °C under a GHSV of 30 000 cm^3^·$g_{\mathrm{cat}}^{-1}$·h^−1^. Han et al. reported a catalyst synthesized via the in situ pyrolysis of La‐doped ZIF‐67 in the mesopores of 2D SBA‐15.^[^ ^21^ ^]^ Benefitting from the double spatially constrained configuration and the synergistic effects of LaCoO_x_, the ammonia decomposition rate of the catalyst obtained reached 100% at 550 °C under a GHSV of 30 000 cm^3^·$g_{\mathrm{cat}}^{-1}$·h^−1^.

In addition to implanting catalytically active species onto support, modification with additional metal compounds as promoters can significantly improve the ammonia decomposition activity and stability of the catalyst via the construction of heterostructures among the active metal‐containing species. Rare‐earth metal oxides (such as LaO_x_ and CeO_x_) have been confirmed as ideal promoters owing to their ability to regulate the alkalinity of the catalyst surface and provide electrons for metal particles,^[^ ^22^ ^]^ which in turn accelerate the recombination and desorption of N atoms, thus enhancing the rate of ammonia decomposition.^[^ ^12^ ^]^ One example is a catalyst of uniformly dispersed transition‐metal nanoparticles loaded on the surface of a La‐promoted MgO carrier, which demonstrated an ammonia decomposition rate that was 25% greater than the La‐free analog.^[^ ^23^ ^]^ In another example, Ru loaded onto Ce/MgAl by the deposition precipitation (DP) method yielded a catalyst that achieved an ammonia conversion rate of 99.5% at 550 °C under a GHSV of 30 000 cm^3^·$g_{\mathrm{cat}}^{-1}$·h^−1^, which has been attributed to an increase in the number of oxygen vacancies and increased basicity caused by the addition of Ce species.^[^ ^24^ ^]^ As a rare‐earth metal oxide with high chemical stability, Y_2_O_3_ has been widely used in catalysis as an accelerator for fixing active centers and maintaining a high dispersion of metal catalysts at high temperatures, which is beneficial for ammonia decomposition reactions.

Typically, a porous structure and promoter are prerequisites for a catalyst with high ammonia decomposition activity and a long lifespan. A porous structure can increase the number of active sites for ammonia adsorption, whereas a promoter can facilitate the recombination and desorption of nitrogen, and inhibit the sintering of metal particles. In this study, a Y_2_O_3_‐modified porous carbon‐encapsuled cobalt catalyst with a uniform distribution of Co nanoparticles (denoted as Y_2_O_3_–Co/NC) was prepared by the pyrolysis of Y_2_O_3_‐doped zeolite imidazolate frameworks for hydrogen production by catalytic ammonia decomposition. In this catalytic system, Co is the active center for the ammonia decomposition reaction, porous nitrogen‐doped carbon (NC) serves as the carrier for dispersing and supporting the Co nanoparticles, and Y_2_O_3_ was used as an accelerator to improve the stability and activity of the catalyst. Benefitting from the complex structure of ZIF‐67, homogeneously dispersed Co nanoparticle‐loaded carbon‐based catalysts were synthesized, and the particle sizes of Co and Y_2_O_3_ originating from the adsorbed Co^2+^ and Y^3+^ were restricted by the N‐doped carbon formed during pyrolysis. The experimental results show that the addition of Y_2_O_3_ significantly improved the activity and stability of the catalyst. The optimized catalyst exhibited an ammonia decomposition rate of 92.3% with a hydrogen production rate of 20.6 mmol·$g_{\mathrm{cat}}^{-1}$·min^−1^ at 550 °C under a GHSV of 20 000 cm^3^·$g_{\mathrm{cat}}^{-1}$·h^−1^. The experimental results and associated theoretical calculations demonstrate that Y_2_O_3_ inhibits the sintering of Co nanoparticles at high temperatures and promotes the recombination and desorption of N atoms on the catalyst surface.

## 2 Results and Discussion

### 2.1 Characterization

**Figure 1:**
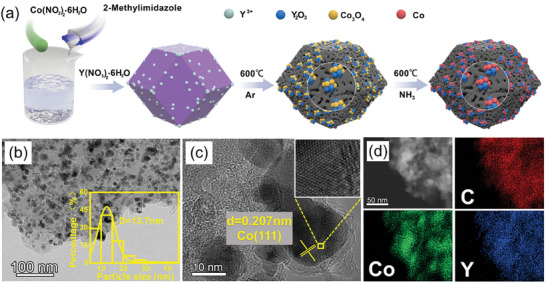
Synthesis and morphology of the designed catalytic materials. (a) Schematic of the synthetic procedure for the xY_2_O_3_–Co/NC catalysts. (b) TEM images and particle size distribution profile, (c) HR‐TEM image and (d) corresponding elemental mapping analysis of 4Y_2_O_3_–Co/NC.

The synthesis of the xY_2_O_3_–Co/NC catalysts is shown schematically in **Figure** **1**a. The complexation of Co^2+^ with 2‐methylimidazole led to the formation of a framework in which metal ions were located at the top of tetrahedral sites and N atoms were positioned in the middle of the tetrahedron sites (denoted as ZIF‐67). The tetrahedral structural units were connected to adjacent Co^2+^ or organic ligands and formed a regular dodecahedral skeleton. After the addition of Y(NO_3_)_3_·6H_2_O to the solution, Y^3+^ ions were adsorbed onto ZIF‐67. During pyrolysis, the organic ligands decomposed into N‐doped carbon supports, which provide additional stabilization to the Co‐based nanoparticles, while the adsorbed Y^3+^ species were oxidized to Y_2_O_3_. Finally, the oxidized cobalt nanoparticles were reduced to Co^0^ by calcination in an ammonia atmosphere; given its thermodynamic stability, Y_2_O_3_ was unaltered.

The morphology of the obtained Y(NO_3_)_3_/ZIF‐67, Co/NC, and Y_2_O_3_–Co/NC catalysts with various Y_2_O_3_ loadings were investigated by scanning electron microscopy (SEM; Figure S1, Supporting Information). Y(NO_3_)_3_/ZIF‐67 exhibited the same dodecahedral structure as ZIF‐67, indicating that the introduction of Y‐containing species did not result in the structural destruction of ZIF‐67 in Y(NO_3_)_3_/ZIF‐67. The SEM images shown in Figure S1b–f (Supporting Information) reveal that a 2D flaky structure was formed in both the synthesized Co/NC catalyst and 4Y_2_O_3_–Co/NC catalyst after sequential annealing under argon and NH_3_ atmospheres. Moreover, their similar morphologies further demonstrate that a doping ratio of Y_2_O_3_ below 8 wt.% has no notable impact on the morphology of the synthesized samples (Figure S1b–f, Supporting Information).

Transmission electron microscopy (TEM) images of Co/NC (Figure S2, Supporting Information) and 4Y_2_O_3_–Co/NC (Figure 1b) show that the Co nanoparticles were uniformly dispersed on the carbon‐based supports with average particle sizes of 14.4 and 13.7 nm, respectively. The decreased particle size after introducing Y_2_O_3_ suggests that the Y_2_O_3_ promoter restricts the growth of the Co nanoparticles, which is beneficial for generating an increased number of active sites. High‐resolution TEM (HR‐TEM) images (Figure S2b, Supporting Information; Figure 1c) reveal that the Co nanoparticles in both the Co/NC and 4Y_2_O_3_–Co/NC catalysts exhibit lattice stripes with a spacing of 0.207 nm, corresponding to the (111) crystal plane of Co metal. Uniformly dispersed Co nanoparticles of small size loaded onto N‐doped carbon are usually regarded as possessing more active sites, which may enhance the catalytic performance of the synthesized materials. However, Y_2_O_3_ lattice stripes were not observed in the 4Y_2_O_3_–Co/NC catalyst, suggesting that the Y_2_O_3_ nanoparticles formed were amorphous or too small to be observed by TEM.^[^ ^22^, ^25^ ^]^ In addition, elemental mapping images show that the distribution of Co in the 4Y_2_O_3_–Co/NC catalyst (Figure 1d) was more uniform than that in the Co/NC catalyst (Figure S2c, Supporting Information), which is attributed to the stabilization effect of the introduced Y_2_O_3_. Additionally, the homogeneous distribution of N in the Co/NC and 4Y_2_O_3_/Co–NC (Figure S3a, Supporting Information) catalysts indicate that N was well retained in the carbon support, and Figure S3b (Supporting Information) shows that O was uniformly distributed within the 4Y_2_O_3_–Co/NC catalyst. Moreover, uniformly dispersed Y confirmed the suitable distribution of Y_2_O_3_ in the 4Y_2_O_3_–Co/NC sample. Based on the energy‐dispersive (EDS) analysis (Figure S3d, Supporting Information), the elemental contents of Y and Co in the 4Y_2_O_3_–Co/NC catalyst were 14.06% and 4.26%, respectively. The selected area electron diffraction (SAED) pattern (Figure S3c, Supporting Information) shows several continuous bright rings, indicating that the prepared 4Y_2_O_3_–Co/NC sample was polycrystalline.

**Figure 2:**
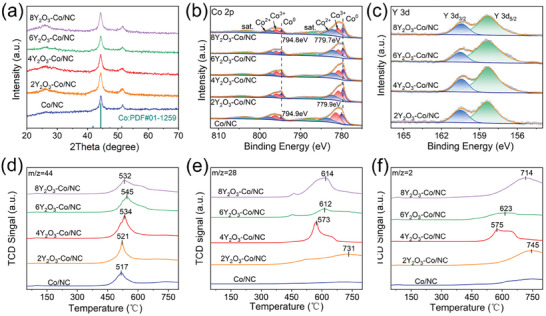
Characterization of samples: (a) XRD patterns; (b) high‐resolution XPS Co 2*p* spectra; (c) high‐resolution XPS Y 3*d* spectra; (d) CO_2_‐TPD curves; (e) N_2_‐TPD curves; and (f) H_2_‐TPD curves of the prepared catalysts.

X‐ray diffraction (XRD) analysis was performed to analyze changes in the crystal structure after the addition of varying quantities of the Y species. The XRD pattern of Y(NO_3_)_3_/ZIF‐67, shown in Figure S4a (Supporting Information), is identical to that of ZIF‐67 minus any impurity peaks, indicating that the crystal structure of ZIF‐67 was retained following the incorporation of Y^3+^. The diffractogram of the sample before reduction with ammonia shows that cobalt exists in the form of Co_3_O_4_ (Figure S4b, Supporting Information). Diffraction peaks are observed at 44.4° and 51.3° in all of the XRD patterns of the Y_2_O_3_‐modified samples, which correspond to the (111) and (200) crystal planes of Co (PDF:01‐1259; **Figure** **2**a), further confirming the successful preparation of Co nanoparticles. However, no diffraction peaks related to the Y_2_O_3_ phase were detected, further suggesting either a small grain size or amorphous Y_2_O_3_, consistent with the HR‐TEM results.

Dinitrogen (N_2_) adsorption‐desorption isotherms were obtained to investigate the effects of Y_2_O_3_ modification on the porous structure of the samples. As shown in Figure S5a (Supporting Information), the isotherms of all samples were type V with type H_3_ hysteresis loops, indicative of the presence of mesopores. Y_2_O_3_ (a rare‐earth oxide) could effectively improve the dispersion of the Co nanoparticles (Table S3, Supporting Information) and limit them to a smaller size. Thus, compared with Co/NC, the introduction of Y_2_O_3_ with a weight content below 4.0 wt.% resulted in a reduced pore diameter (Figure S5b, Supporting Information) and increased specific surface area (Table S1, Supporting Information), thus affording improved adsorption of ammonia and subsequently enhanced exposure of the active sites for catalytic reactivity. Indeed, the Y_2_O_3_‐modified catalyst facilitated the gradual dehydrogenation of NH_x_ on the surface, resulting in improved catalytic activity toward the thermal decomposition of ammonia. However, the specific surface area of the catalyst decreased significantly as the Y content increased to 6 and 8 wt.%, probably due to pore blockage caused by the redundant addition of Y_2_O_3_.^[^ ^26^ ^]^

The Raman spectra of the synthesized samples are displayed in Figure S5c (Supporting Information). The D‐band (1347 cm^−1^) and G‐band (1580 cm^−1^) characteristic peaks of carbon suggest the presence of a graphitic carbon layer in all tested samples.^[^ ^27^ ^]^ Moreover, the increased peak intensity ratios of the D‐ and G‐bands (I_D_/I_G_) with increasing Y content is evidence of increased formation of defective carbon caused by the replacement of Co^2+^ sites in ZIF‐67 with Y^3+^ during high‐temperature calcination. The presence of defects in the catalyst support has been reported to be beneficial for ammonia decomposition owing to the increased number of active sites on the catalyst surface.^[^ ^21^ ^]^

X‐ray photoelectron spectroscopy (XPS) was used to determine the chemical environment and electronic structure of elements near the catalyst surface, which are important factors affecting the ammonia decomposition reactivity. The XPS survey spectra of the target catalysts (Figure S6a, Supporting Information) show the coexistence of Co, Y, C, N, and O. Two main peaks in the high‐resolution Co 2*p* XP spectra (Figure 2b) exhibit spin‐orbital splitting and correspond to the Co 2*p*_3/2_ and Co 2*p*_1/2_ orbitals, respectively, while two pairs of broad peaks are assigned to satellite peaks.^[^ ^28^ ^]^ The Co 2*p*_1/2_ peaks can be deconvoluted into three peaks at 797.6–797.8, 796–796.2, and 794.8–794.9 eV, corresponding to Co^3+^, Co^2+^, and Co^0^ species, respectively. Similarly, the Co 2*p*_3/2_ peaks were deconvoluted into peaks corresponding to three species of Co^3+^, Co^2+^, and Co^0^ at 782.3–783.7, 780.8–781.4, and 779.7–779.9 eV, respectively. Compared to the Co/NC catalyst, the characteristic Co^0^ peak in the Co 2*p*_1/2_ and Co 2*p*_3/2_ regions of the Y_2_O_3_‐doped Co/NC catalysts shifted to lower binding energy by 0.1 and 0.2 eV, respectively, suggesting that Y_2_O_3_ donates electron density to Co, which is advantageous as the resulting electron‐rich Co on the catalyst surface has been reported to be beneficial for the ammonia decomposition reaction.^[^ ^29^ ^]^ The distribution of the different chemical states of Co in the prepared samples is summarized in Table S2 (Supporting Information), and show that Y_2_O_3_ doping led to an increase in Co° content on the catalyst surface. The high‐resolution O 1*s* XPS profiles, shown in Figure S6b (Supporting Information), were deconvoluted into two peaks at 531.8 and 529.9 eV, corresponding to chemisorbed oxygen (O_ads_) and lattice oxygen (O_latt_), respectively. The deconvoluted peaks for C─C (284.8 eV), C─N (285.1–285.5 eV) and C─O (284.6–284.1 eV) binding environments in the high‐resolution C 1*s* XP spectra (Figure S6c, Supporting Information), together with the existence of pyridine‐N, pyrrolic‐N, and graphitic‐N found in the high‐resolution N 1*s* XP spectra (Figure S6d, Supporting Information) demonstrate that the N‐atoms in ZIF‐67 remain in the carbon matrix during the annealing process.^[^ ^30^ ^]^ In the high‐resolution Y 3*d* XP spectra (Figure 2c), the peak at 158.1 eV for Y 3*d*_5/2_ corresponds to the Y─O bond in Y_2_O_3_, and the peak at 160.4 eV for Y 3*d*_3/2_ is related to the Y─(OH)_x_ or Y─CO bonds;^[^ ^25^ ^]^ the formation of Y─(OH)_x_ or Y─CO bonds is a result of absorption of moisture or carbon dioxide from air by Y_2_O_3_. In addition, the relative Y 3d_5/2_:Y 3d_3/2_ peak height ratio was 3:2, which is close to the theoretical stoichiometric volume of Y_2_O_3_, further indicating the existence of Y in the form of Y_2_O_3_.

To determine the dispersion of the Co nanoparticles in the prepared catalysts, CO pulse chemisorption was conducted, and the results are shown in Table S3 (Supporting Information). The degree of dispersion of the Co nanoparticles in the Y_2_O_3_‐doped Co/NC catalyst was higher than that in the Co/NC catalyst (2.22%), and 4Y_2_O_3_–Co/NC showed the highest degree of dispersion (2.47%), indicating that 4Y_2_O_3_–Co/NC possesses the largest number of exposed active sites and may exhibit optimal thermal catalytic performance in the ammonia decomposition reaction. The increased degree of dispersion of Co nanoparticles following the introduction of Y_2_O_3_ could result from the enhanced interaction between the Co nanoparticles and the support, leading to the formation of relatively small Co nanoparticles.^[^ ^31^, ^32^ ^]^ However, the degree of Co nanoparticle dispersion decreased when the Y content exceeded 4 wt.% due to partial coverage of the Co surface by Y_2_O_3_, evidenced by the reduced contact with CO during the CO pulse chemisorption experiments.

The surface basicity of the catalyst is another important factor that affects ammonia decomposition, and CO_2_ temperature‐programmed desorption (TPD) experiments were performed to determine the surface basicity of the prepared catalysts.^[^ ^33^ ^]^ As shown in Figure 2d, the Co/NC and Y_2_O_3_‐doped Co/NC catalysts only exhibit one CO_2_ desorption peak located in the high‐temperature region (> 510 °C), indicative of the strong basicity of the catalyst surface.^[^ ^34^, ^35^ ^]^ Moreover, the increased temperature for CO_2_ desorption following Y_2_O_3_‐doping indicates that the introduction of Y_2_O_3_ can improve the surface basicity, which can promote the recombination and desorption of N atoms on the surface of the catalyst through enhanced electron transfer, thereby accelerating the efficiency of hydrogen production by ammonia decomposition.^[^ ^36^, ^37^ ^]^ This hypothesis was further confirmed by the N_2_ temperature‐programmed desorption (N_2_‐TPD) results (Figure 2e). Compared to the sample of Co/NC, which has no desorption peaks in the tested temperature range, the Y_2_O_3_‐doped samples show a strong N_2_ desorption peak above 550 °C, indicating that the introduction of Y_2_O_3_ can effectively reduce the Co─N binding energy and can accordingly promote the reorganization of N‐atoms and desorption of N_2_. Among the samples tested, 4Y_2_O_3_–Co/NC showed the lowest desorption temperature (573 °C) and therefore the weakest Co─N binding energy. In addition, H_2_ desorption from the catalyst surface on a suitable timescale is an important factor in the ammonia decomposition reaction kinetics because of the reduced coverage of active sites by H_2_.^[^ ^37^, ^38^ ^]^ Similarly, the H_2_ temperature‐programmed desorption (H_2_‐TPD) results (Figure 2f) revealed that the H_2_ desorption peak for the Y_2_O_3_‐doped sample exhibited a higher peak intensity and lower desorption temperature than those of the Co/NC catalyst, indicating that Y_2_O_3_‐doping can also effectively improve H_2_ desorption from the catalyst surface, facilitating NH_3_ dehydrogenation and improving catalytic activity.

### 2.2 Catalytic Ammonia Decomposition Activity

**Figure 3:**
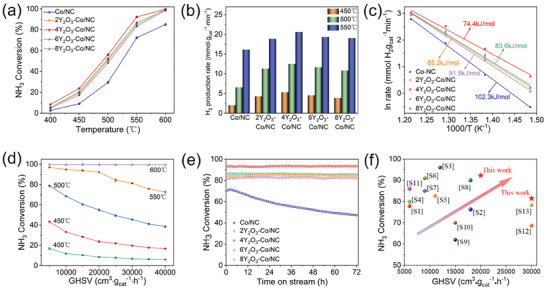
Catalytic activity toward ammonia decomposition. (a) Ammonia conversion as a function of temperature at a GHSV of 20 000 cm^3^·$g_{\mathrm{cat}}^{-1}$·h^−1^. (b) Rate of hydrogen formation for the prepared samples. (c) The apparent activation energy (*E_a_*) for the prepared samples. (d) The effect of GHSV on NH_3_ conversion on the 4Y_2_O_3_–Co/NC catalyst at varying temperatures. (e) Stability results measured at 550 °C and a GHSV of 20 000 cm^3^·$g_{\mathrm{cat}}^{-1}$·h^−1^. (f) Comparison of the catalytic activity in this work with catalysts reported in the literature at 550 °C.

The promotional effects of different rare‐earth metal oxides on the Co/NC catalyst were investigated, and the results are plotted in Figure S7a (Supporting Information). The effects of CeO_2_‐, La_2_O_3_‐, and Pr_11_O_6_‐doping on catalysis were significantly less than that of Y_2_O_3_, suggesting that the complex catalytic system containing Y_2_O_3_, Co, and NC is optimal for ammonia decomposition. Therefore, the activity of the xY_2_O_3_–Co/NC catalyst was further investigated, and detailed ammonia decomposition properties of the prepared catalysts were evaluated (**Figure** **3**). Evidently, temperature‐dependent ammonia conversion displays S‐type curves for all catalysts, and the introduction of Y species improved the catalytic activity toward ammonia decomposition within the tested temperature range of 400–600 °C (Figure 3a). Moreover, the effect of Y species content exhibits non‐monotonic behavior. In particular, ammonia conversion initially increases then decreases with increasing Y content above 4 wt.%. The 4Y_2_O_3_–Co/NC sample reached the highest conversion rate of 56.1% at 500 °C and 92.3% at 550 °C. Results from inductively coupled plasma‐optical emission spectroscopy (ICP‐OES) revealed that the Co content only decreased slightly with increasing Y content (Table S4, Supporting Information), indicating that the improved catalytic activity toward ammonia decomposition originates from the introduced Y species. Similarly, as shown in Figure 3b, 4Y_2_O_3_–Co/NC exhibits the best hydrogen production rate, reaching 20.6 mmol·$g_{\mathrm{cat}}^{-1}$·min^−1^ at 550 °C. This was further evidenced by the apparent activation energies (*E_a_*) for the synthesized catalysts, shown in Figure 3c, in which 4Y_2_O_3_–Co/NC exhibits the lowest *E_a_* value of 74.4 kJ·mol^−1^. In addition, the turnover frequency (TOF) of the Y_2_O_3_‐doped Co/NC catalysts was significantly higher than those of the Co/NC catalysts at 450 °C (Figure S7b, Supporting Information); the TOF of 4Y_2_O_3_–Co/NC at 450 °C reached 3.58 s^−1^, which was more than twice that of the Co/NC catalyst.

Ammonia decomposition over the 4Y_2_O_3_–Co/NC catalyst under different GHSVs was investigated to determine the influence of GHSV on ammonia conversion (Figure 3d). Ammonia conversion at 600 °C using the 4Y_2_O_3_–Co/NC catalyst was > 99% even if the GHSV reached 40 000 cm^3^·$g_{\mathrm{cat}}^{-1}$·h^−1^ due to fast reaction kinetics. However, the ammonia conversion rate decreased with increasing GHSV once the reaction temperature was reduced to below 600 °C. The reaction kinetics become slower with decreasing temperature, and the reduced time of adsorption with increasing GHSV may not guarantee efficient conversion of ammonia, leading to reduced conversion rates.^[^ ^36^ ^]^

Catalyst stability is of concern for practical applications. As a result, stability tests were conducted at 550 °C under a GHSV of 20 000 cm^3^·$g_{\mathrm{cat}}^{-1}$·h^−1^ for 72 h. As shown in Figure 3e, the ammonia conversion rates for all Y_2_O_3_‐doped Co/NC catalysts remained stable within the testing time, indicating excellent stability. In comparison, the ammonia decomposition rate for the Co/NC catalyst decreased by ≈20% after 72 h, which is attributed to the reduced number of active sites induced by the agglomeration of Co nanoparticles on the catalyst surface.^[^ ^22^ ^]^ Thus, Y_2_O_3_‐doping can significantly improve catalyst stability by suppressing the sintering of Co nanoparticles during high‐temperature reactions. Moreover, as shown in Figure 3f and Table S5 (Supporting Information), the ammonia decomposition performance for the 4Y_2_O_3_–Co/NC catalyst is superior to most non‐noble metal catalysts reported recently, effectively reducing the energy consumption for hydrogen production by ammonia decomposition and rendering this approach promising as an efficient energy conversion technology for multi‐scenario applications.

### 2.3 Morphological and Structural Stability

**Figure 4:**
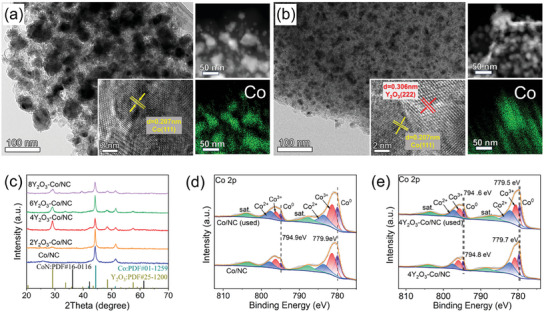
Morphological and structural characterization of samples after stability testing. (a) TEM images and corresponding elemental mapping of the synthesized Co/NC and (b) 4Y_2_O_3_–Co/NC catalysts. The insets in (a) and (b) are HR‐TEM images with marked lattice fringes and particle size distribution profiles. (c) XRD patterns of the catalysts post‐catalysis. (d) Co 2*p* XP spectra of the Co/NC catalyst pre‐ and post‐catalysis. (e) Co 2*p* XP spectra of the 4Y_2_O_3_–Co/NC catalyst pre‐ and post‐catalysis.

Following stability testing at 550 °C under a GHSV of 20 000 cm^3^·$g_{\mathrm{cat}}^{-1}$·h^−1^ for 72 h, the morphology of the samples was investigated to determine the influence of Y_2_O_3_‐doping on sample stability. The SEM images (Figure S8, Supporting Information) revealed that the size of the metal particles in the Y_2_O_3_‐doped Co/NC samples changed slightly post‐catalysis, whereas significant aggregation of metal particles was observed on the surface of the Co/NC catalyst. This phenomenon was also clearly observed in the TEM images (**Figure** **4**a,b). Specifically, as shown in the particle size distribution histograms derived from TEM images, the average particle size of 4Y_2_O_3_–Co/NC increased slightly from 13.7 to 16.3 nm following stability testing, whereas it increased significantly from 14.4 to 26.2 nm for Co/NC. Furthermore, N is uniformly distributed in both the Co/NC and 4Y_2_O_3_–Co/NC catalysts, suggesting the absence of cobalt nitride (Figures S9 and S10a, Supporting Information). Elemental mapping images further confirm that Y_2_O_3_ doping can effectively inhibit the sintering of Co nanoparticles during thermal ammonia decomposition process, as evidenced by the aggregation of Co nanoparticles in Co/NC sample and the uniform distribution both of Co and Y (Figure S10b, Supporting Information) in 4Y_2_O_3_–Co/NC. In tThe HR‐TEM images of the Co/NC and 4Y_2_O_3_–Co/NC catalysts post‐stability testing contained lattice fringes with spacings of 0.207 nm, corresponding to the Co(111) crystal plane, indicating that the phase of Co remained unchanged during the reaction. Lattice fringes with a spacing of 0.309 nm, corresponding to the (222) crystal plane of Y_2_O_3_, were also observed around the Co(111) lattice fringes of the used 4Y_2_O_3_–Co/NC sample. The appearance of Y_2_O_3_ lattice fringes and the slight change in particle size are suggestive of the structural reconstruction of the 4Y_2_O_3_–Co/NC catalyst during ammonia decomposition.

The XRD patterns of the samples following stability testing are displayed in Figure 4c. Compared to the Co/NC catalyst, the Y_2_O_3_‐doped samples exhibit new diffraction peaks at 29.2°, 37.9°, and 48.5° following stability testing, which correspond to the (222), (420), and (440) crystal planes of Y_2_O_3_ (PDF:25‐1200), consistent with the HR‐TEM results. Moreover, the N_2_ adsorption/desorption isotherms (Figure S11a, Supporting Information) revealed that the mesoporous structure of all the samples was maintained post‐stability testing. Compared to the fresh samples, the increased pore size and decreased specific surface area following stability testing (Figure S11b and Table S1, Supporting Information) could be attributed to the agglomeration of Co nanoparticles on the Co/NC catalyst surface, and structural reconstruction induced the formation of Y_2_O_3_ nanoparticles in the Y_2_O_3_‐doped samples. However, variations in the average pore size and specific surface area in the Y_2_O_3_‐doped samples were less pronounced than those of the Co/NC sample after stability testing, further demonstrating the inhibitory effect of Y_2_O_3_ on the agglomeration of Co nanoparticles.

The electronic states on the surface of the Co nanoparticles in the Co/NC and 4Y_2_O_3_–Co/NC catalysts following stability testing were further investigated using high‐resolution Co 2*p* XPS. As shown in Figure 4d, no change in the binding energy of Co^0^ was observed in the Co 2*p* spectra of the Co/NC sample before and after the stability test. However, the binding energy of Co^0^ shifts negatively by 0.2 eV in the Co 2*p* XPS profiles of 4Y_2_O_3_–Co/NC (Figure 4e) following the stability test, indicating that Y_2_O_3_ can provide electrons to Co during the ammonia decomposition process; Table S2 (Supporting Information) shows that the Co° content in both the Co/NC and 4Y_2_O_3_–Co/NC catalysts remained unchanged after the stability tests. The electron‐rich surface of the Co nanoparticles has been shown to be beneficial for the breakage of the N─H bond of ammonia, thus promoting the ammonia decomposition activity of the catalyst.^[^ ^39^ ^]^ The N 1*s* XP spectrum of the Co/NC and 4Y_2_O_3_–Co/NC catalysts after stability testing are shown in Figure S11c,d (Supporting Information). The spectra exhibit no notable changes, confirming the absence of cobalt nitride formation.

### 2.4 Density Functional Theory Calculations

**Figure 5:**
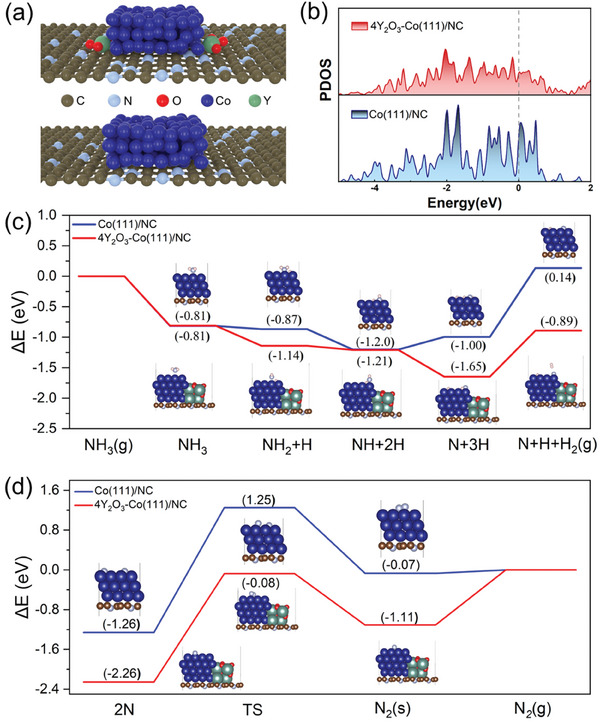
Theoretical calculation. (a) Atomic model for Co(111)/NC and 4Y_2_O_3_–Co(111)/NC. (b) Projected density of states (PDOS) in the Co(111)/NC and 4Y_2_O_3_–Co(111)/NC models. Energy profiles of (c) the elementary reaction steps during the dehydrogenation of NH_x_ and (d) the associated desorption of N atoms on Co(111)/NC and 4Y_2_O_3_–Co(111)/NC.

To elucidate the enhancement in ammonia decomposition activity for the Y_2_O_3_‐doped Co/NC catalysts, density functional theory (DFT) calculations were performed to determine the energy required for each elementary reaction step on Co(111)/NC and 4Y_2_O_3_–Co(111)/NC. Generally, the decomposition reaction mechanism includes N─H bond cleavage for dehydrogenation and desorption of N_2_. Models of the atomic structures of Co(111)/NC and 4Y_2_O_3_–Co(111)/NC during ammonia decomposition were constructed based on their proposed structures (**Figure** **5**a), as shown in Figures S12 and S13 (Supporting Information), respectively. The projected density of electronic states (PDOS), shown in Figure 5b, reveals that the introduction of Y_2_O_3_ alters the electronic states near the Fermi level, thus significantly enhancing the interaction between the substrate and Co atoms.^[^ ^40^ ^]^

The corresponding energy profiles of the optimized intermediate structures during the dehydrogenation steps are shown in Figure 5c, and detailed calculations related to the energies required for the two samples are listed in Table S6 (Supporting Information). The adsorption energies of the NH_2_, NH, N, and H intermediates on the 4Y_2_O_3_–Co(111)/NC surface were more negative than those on the Co(111)/NC surface, indicating more favorable adsorption on 4Y_2_O_3_–Co(111)/NC. In addition, the adsorption of all intermediates on the surfaces of both samples was exothermic, and the reaction energies for the four elementary reactions during the dehydrogenation process demonstrate that the elementary reaction H^*^ + H^*^→H_2_ + 2^*^ requires more energy than the other three steps, as evidenced by large positive reaction energies for both samples (Table S6, Supporting Information). Moreover, the reaction energy of the highest energy‐consuming step is ≈0.37 eV lower for 4Y_2_O_3_–Co(111)/NC than for Co(111)/NC, indicating that Y_2_O_3_‐doping can enhance dehydrogenation of ammonia, consistent with the H_2_‐TPD measurements (Figure 2f).

The energy profiles associated with the desorption of N atoms and the detailed results for the two samples are displayed in Figure 5d and Table S7 (Supporting Information), respectively. The recombination and desorption energy barriers of N atoms on the catalyst surface are 2.51 and 2.18 eV for Co/NC and 4Y_2_O_3_–Co(111)/NC, respectively. Compared to the dehydrogenation process discussed above, the recombination and desorption of N atoms exhibited the highest activation energy for both samples, suggesting that the recombination and desorption of N atoms are the rate‐limiting steps of the thermal ammonia decomposition reaction. In addition, the energy barrier for the recombination and desorption of N atoms on the surface of 4Y_2_O_3_–Co(111)/NC is ≈0.33 eV lower than that on the surface of Co(111)/NC, indicating that both processes are more facile on the surface of 4Y_2_O_3_–Co(111)/NC, consistent with the N_2_‐TPD results (Figure 2e). According to the characterization results and DFT calculations, Y_2_O_3_‐doping can enhance the recombination and desorption of N atoms and the gradual dehydrogenation of NH_x_ on the catalyst surface, and inhibits the sintering of Co nanoparticles, which in turn enhances the performance of the synthesized catalyst.

### 2.5 Operando TEM Characterization

**Figure 6:**
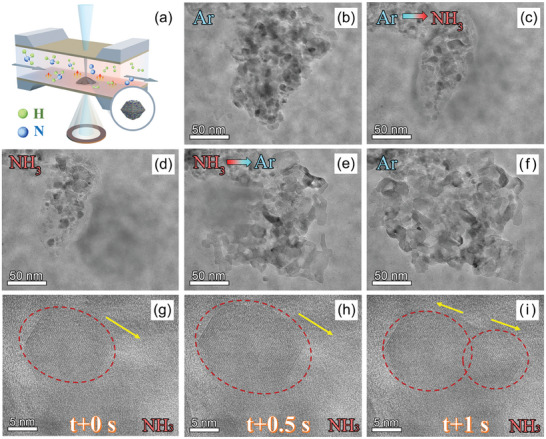
Operando TEM characterization. (a) Schematic of the experimental setup for operando TEM characterization. (b–f) Operando TEM images of the microstructural and morphological changes of 4Y_2_O_3_–Co/NC in different reaction atmospheres, with varied timescales, at 500 °C. (g–i) Operando HR‐TEM images of the growth and division of the Co(111) lattice stripe in 4Y_2_O_3_–Co/NC under an ammonia atmosphere at 500 °C.

Operando TEM was conducted to directly investigate the thermal decomposition of ammonia using the synthesized 4Y_2_O_3_–Co/NC catalyst. A schematic of the experimental setup, reaction chamber, and the applied chips are displayed in **Figures** **6**a and S14 (Supporting Information). The experiment was first carried out in an argon atmosphere at 500 °C. As displayed in the recorded Movie S1 (Supporting Information) and Figure 6b, no morphological and structural changes were observed, indicative of the superior stability of 4Y_2_O_3_–Co/NC catalyst under an electron beam at 500 °C. After switching the reaction atmosphere from argon to ammonia, Co nanoparticle migration and a significant change in the morphology of the carbon carriers were clearly observed, as shown in Figure 6c,d. Moreover, as the reaction proceeded, the morphology of some of the Co nanoparticles and the carbon carriers became fuzzy, and the loaded catalyst moved rapidly (Movie S2, Supporting Information). This suggests that the loaded catalysts were transformed into a new liquid form during the ammonia decomposition reaction. This is also supported by the literature, in which the transformation of metal nanoparticles into a liquid‐state at temperatures far below the melting point of the respective metal have been reported.^[^ ^41^ ^]^ When the reaction atmosphere was switched back to argon (Figure 6e,f), the liquid‐like movement of the catalysts gradually slowed and eventually stopped as a solid‐state was again accessible. In addition, the in situ recorded HR‐TEM images of the Co nanoparticles during ammonia thermal decomposition revealed that the loaded 4Y_2_O_3_–Co/NC catalyst underwent a dynamic reconstruction process entailing the cyclic growth and splitting of the Co(111) lattice stripes (Figure 6g–i), which was complete within 2 s (Movie S3, Supporting Information). According to the widely accepted mechanism for the ammonia decomposition reaction, the splitting of Co(111) lattice stripes may be related to the destruction of the lattice caused by the combination of N atoms and Co nanoparticles during ammonia decomposition, while the emergence of the Co(111) lattice stripes is related to the desorption of N atoms from the catalyst surface and reorganization of N_2_ to restore the Co lattice. Combined with the HR‐TEM images of the 4Y_2_O_3_–Co/NC catalyst following the stability tests, as shown in Figure 4b, the 4Y_2_O_3_–Co/NC catalyst are concluded to participate in the ammonia decomposition reaction, and the slight change in the size of the Co nanoparticles originates from particle sintering, which is inhibited by rare earth oxides, as evidenced by the appearance of the Y_2_O_3_ lattice and dynamic reconstruction, which entails growth and splitting of the Co nanoparticles during thermal ammonia decomposition.^[^ ^42^ ^]^

## 3 Conclusion

In summary, catalysts of Y_2_O_3_‐stabilized Co loaded onto porous N‐doped carbon (Y_2_O_3_–Co/NC) were successfully synthesized by pyrolysis of Y(NO_3_)_3_‐modified ZIF‐67 under an argon atmosphere, followed by sintering in a reductive NH_3_ environment. The Y_2_O_3_ introduced onto the catalyst support prevented further growth of the Co nanoparticles, and their relatively small size enhanced the number of catalytically active sites. Furthermore, due to its electron donor ability, Y_2_O_3_ enhanced the surface alkalinity, which in turn improved the recombination and desorption of nitrogen atoms. Moreover, the observed electron transfer from the Y species to the Co atoms led to an electron‐rich Co nanoparticle surface. Accordingly, catalytic dehydrogenation of reaction intermediates (NH_x_) was improved, and the recombination and desorption of hydrogen atoms were accelerated. In addition, the observed dynamic reconstruction of the catalyst during ammonia thermal decomposition by *operando* characterization was indicative of enhanced catalytic stability, rendering this catalytic system widely applicable for large‐scale implementation. Based on the results reported herein, the synthesis of non‐noble metal catalysts with exceptional thermal catalytic performance was feasible, which incorporated small nanoparticles and exhibited excellent cycling stability as a result of the presence of rare‐earth oxides.

## 4 Experimental Section

### Catalyst Synthesis

A 100 mL aqueous solution containing 2‐methylimidazole (2.952 g, XX mol) was slowly added to an aqueous solution of Co(NO_3_)_2_·6H_2_O (1.74 g, XX mol in 100 mL ionized water) with magnetic stirring. After stirring for 2 h, Y(NO_3_)_3_·6H_2_O (0.654 g, XX mol) was added to the solution with continuous stirring for 4 h. The obtained precursor was separated by centrifugation, washed extensively with deionized water and dried overnight at 60 °C, providing Y(NO_3_)_3_/ZIF‐67. To obtain 4Y_2_O_3_–Co/NC, the precursor was calcined at 600 °C for 2 h with a ramp rate of 3 °C·min^−1^ under an argon atmosphere, then annealed in an ammonia atmosphere for 2 h. The control samples were synthesized according to the same procedure, however with the addition of varying amounts of Y(NO_3_)_2_·6H_2_O (0, 0.327, 0.981, and 1.308 g). Samples were denoted as xY_2_O_3_–Co/NC, where x refers to the weight percentage of yttrium.

### Catalyst Characterization

Powder XRD was performed on an X'Pert PRO MPD operating at 40 kV and 100 mA with Cu Kα radiation (λ = 1.54056 Å). X‐ray photoelectron spectroscopy (XPS) measurements were performed on a Thermo ESCALAB 250XI spectrometer calibrated with C 1*s* binding energy at 284.8 eV. The Co and Y contents of the catalysts were determined using ICP‐OES (PerkinElmer 8300). Scanning electron microscopy (SEM) was conducted using a Zeiss Sigma 300 microscope. High‐resolution TEM (HR‐TEM) was performed using an FEI Tecnai F20 microscope (FE‐TEM) at an acceleration voltage of 200 kV. The surface areas and pore size distributions of the catalysts were determined using a Micromeritics ASAP 2020 surface area analyzer. Raman spectra were recorded using a LabRam HR Evolution spectrometer.

To further analyze the basic sites of the catalysts, CO_2_ temperature‐programmed desorption (CO_2_‐TPD), N_2_‐TPD, and H_2_‐TPD experiments were performed on an AutoChem1 II 2920 instrument equipped with a thermal conductivity detector (TCD) to determine gas consumption. CO pulse chemisorption was performed to measure the surface area of exposed Co. Further details on the catalyst characterization are provided in the Supporting Information.

For characterization by operando transmission electron microscopy (transmission electron microscope with a Tsitan Cubed Themis G2 300 Probe Corrector), the 4Y_2_O_3_–Co/NC catalyst was first dispersed in an ethanol solution by ultrasonication, and the supernatant droplets were then added to a DENS solution chip. After drying, the chip that contained the catalyst was assembled into an in situ TEM gas‐phase holder (Climate S3^+^ system), which was then placed into a transmission electron microscope (Talos F200X) equipped with a high‐speed camera (Ceta 2, FEI) to record images and videos of the in situ catalytic reaction at an acceleration voltage of 200 kV. Prior to the in situ experiment, the entire gas path system was cleaned with argon for 30 min, after which the catalyst was heated to 500 °C at a ramp rate of 30 °C·min^−‐1^ under an argon atmosphere. The gas was switched to ammonia at 500 °C, and the alterations to the catalyst surface were recorded with a high‐speed camera during the reaction.

### Catalytic Measurements

Catalytic ammonia decomposition was measured in a quartz tubular flow reactor with an inner diameter of 8 mm. Catalyst (100 mg) was loaded into the reactor and pure gaseous NH_3_ was introduced at atmospheric pressure with a GHSV of 20 000 cm^3^·$g_{\mathrm{cat}}^{-1}$·h^−1^. Before the test, the catalyst was reduced with pure NH_3_ at 550 °C for 1 h. The ammonia conversion was measured in the temperature range 400—600 °C at a step rate of 50 °C, and each test temperature was maintained for 1 h to ensure temperature stability. Online analysis of the discharged H_2_ was performed using a gas chromatograph equipped with a TCD (SHIMADZU GC‐2030). The gas concentration was measured using an external standard method, constructing a calibration curve using standard gases of varying concentrations (Figure S15, Supporting Information). To evaluate the high‐temperature stability of the catalyst, the reaction temperature was maintained at 550 °C for 72 h. The rate of NH_3_ conversion (X_NH3_) and H_2_ production (R_H2_), TOFs, and apparent activation energy (*E_a_*) were calculated using the following equations:

(1) $$X_{NH_{3}}=\frac{2N_{H_{2}}}{3-2N_{H_{2}}}\times 100\%$$

where $X_{{NH}_{3}}$ is the conversion rate of NH_3_ and $N_{H_{2}}$ is the H_2_ concentration in the generated gas;

(2) $$R_{H_{2}}=\frac{\frac{3}{2}\times V_{NH_{3}}\times X_{NH_{3}}}{m_{\mathrm{cat}}}$$

where m_cat_ is the mass of the catalyst in the reactor and $V_{NH_{3}}$ is the total molar flow rate;

(3)

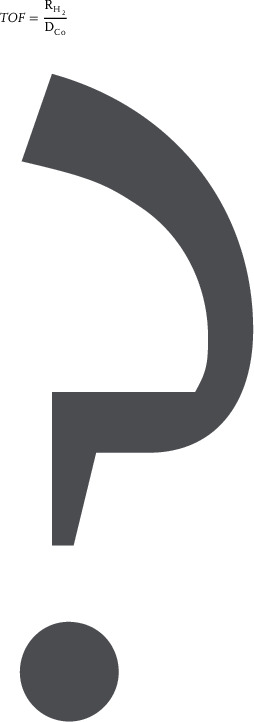

where D_Co_ is the Co dispersion; and the Arrhenius equation,

(4) $$k=\mathrm{Aexp}\left(\frac{-E_{a}}{\mathrm{RT}}\right)$$

where, k is the reaction rate constant, A is the frequency factor, R is the gas constant, and T is the absolute temperature.

### Statistical Analysis

The catalyst activity data for ammonia decomposition were obtained by conducting three repeated tests whereby the average value has been reported. The XRD data were analyzed using MDI Jade6 software. The XPS data were analyzed using Avantage software. The spacing between lattice fringes in the TEM images was measured using Digital Micrograph software, and the average particle size of the Co nanoparticles was calculated using ImageJ software.

## Conflict of Interest

The authors declare no conflict of interest.

## Author Contributions

Y.Z. performed investigation, data curation, formal analysis, writing – original draft, writing – review, and editing. Q.L. performed validation, data curation, methodology, writing – review, and editing. X.H. performed software, calculation. W.X. performed investigation, formal analysis, writing – review, and editing. H.T. performed investigation, data curation, writing – review, and editing. W.T. performed validation, formal analysis, writing – review, and editing. S.W. performed validation, formal analysis, writing – review, and editing. H.P. performed funding acquisition, formal analysis, investigation, methodology, writing – review, and editing. H.T. performed funding acquisition, supervision, acquired resources, writing – review, and editing. H.Z. performed conceptualization, methodology, funding acquisition, acquired resources, supervision, writing – review, and editing.

## Supporting information



Supporting Information

Supplemental Movie 1

Supplemental Movie 2

Supplemental Movie 3

## Data Availability

The data that support the findings of this study are available from the corresponding author upon reasonable request.
