# Supplementary material for: Dynamic Reconstruction of Yttrium Oxide‐Stabilized Cobalt‐Loaded Carbon‐Based Catalysts During Thermal Ammonia Decomposition
Source: Adv Sci (Weinh). 2024 Sep 24;11(43):2406659. doi: 10.1002/advs.202406659 (PMC11578298; doi:10.1002/advs.202406659)
Supplement: Supplementary file 1 — Supporting Information [file ADVS-11-2406659-s001.docx]

**Supporting Information**

**Dynamic Reconstruction of Yttrium Oxide-Stabilized Cobalt-Loaded Carbon-Based Catalysts during Thermal Ammonia Decomposition**

*Yi Zhu ^a, b^, Hongfei Pan^*, a, b^, Qi Li ^a, b^, Xiege Huang ^c^, Wei Xi ^d^, Haibo Tang ^b^,Wenmao Tu ^a^, Shihao Wang ^a, b^, Haolin Tang^*, a, b^, Haining Zhang^*, a, b ,e^*

a State Key Laboratory of Advanced Technology for Materials Synthesis and Processing, Wuhan University of Technology, Wuhan 430070, China

b R&D Center of Materials and Stack Technology for Fuel Cell, National Energy Key Laboratory for New Hydrogen-Ammonia Energy Technologies, Foshan Xianhu Laboratory, Foshan 528200, China

c Hubei Key Laboratory of Theory and Application of Advanced Materials Mechanics, Wuhan University of Technology, Wuhan 430070, China

d School of Chemical Engineering and Technology, Tianjin University, Tianjin 300382, China.

e Hubei Key Laboratory of Fuel Cell, Wuhan University of Technology, Nr. 122 Luoshi Rd., Wuhan 430070, China

**1. Characterization details**

X-ray powder diffraction (XRD): Samples were scanned at a rate of 0.01° step^-1^ from 2θ = 10° ~ 80° with a scan time 1 s step^−1^.

CO_2_ temperature programmed desorption (CO_2_-TPD): Prior to experiment, 50 mg of catalyst was pre-treated in helium atmosphere (50 mL⋅min^-1^) at 300 ℃ for 1 h. After cooling to 50 ℃ in He flow (50 mL⋅min^-1^), the sample was exposed to a 10% CO_2_/Ar flow (50 mL⋅min^-1^) at 50 ℃ for 1 h, followed by Ar flow (50 mL⋅min^-1^) for 1 h. Finally, the catalyst was heated at 10 ℃⋅min^-1^ to 800 ℃.

Temperature-programmed desorption of N_2_ (N_2_-TPD): Before the N_2_-TPD analysis, 50 mg of the catalyst was pre-treated at 300 ℃ for 1 h and then cooled to 50 ℃ under a He atmosphere with a flow rate of 50 mL min^-1^. After that, the gas flow was changed to 10% N_2_/Ar for 1 h at the similar flow rate, followed by Ar flow (50 mL⋅min^-1^) for 1 h. Finally, the catalyst was heated at 10 ℃ min^-1^ to 800 ℃.

Temperature-programmed desorption of H_2_ (H_2_-TPD): Before the H_2_-TPD analysis, 50 mg of the catalyst was pre-treated at 300 ℃ for 1 h and then cooled to 50 ℃ under a He atmosphere with a flow rate of 50 mL min^-1^. After that, the gas flow was changed to 10% H_2_/Ar for 1 h at the similar flow rate, followed by Ar flow (50 mL⋅min^-1^) for 1 h. Finally, the catalyst was heated at 10 ℃ min^-1^ to 800 ℃.

CO pulse chemisorption: Prior to CO pulse chemisorption experiment, 50 mg of the catalyst was pre-treated at 300 ℃ for 1 h and then cooled to 50 ℃ under a He atmosphere with a flow rate of 50 mL min^-1^. After that, 1.0 mL pulses of 10.12% CO/He mixture gas were periodically injected into the reduced sample until the sample was saturated adsorbed by CO. Co dispersion was calculated via assuming an adsorption stoichiometry of 1:1 for CO/Co.

**2. Computational details**

***Methods:*** DFT calculations were performed using the projected augmented wave (PAW) method as implemented in the Vienna *ab initio* Simulation Package (VASP). The generalized gradient approximation (GGA) describes electronic exchange-correlation with Perdew-Burke-Ernzerhof (PBE) functional. The plane wave basis was set as 600 eV, and all calculations used the gamma-centered Monkhorst-Pack scheme. The convergence for the electronic self-consistent field and force criterion were set to $1\times{10}^{-6}$ eV and $1\times{10}^{-2}$ eV/Å, respectively. Transition states were estimated using the climbing image nudged elastic band method (CI-NEB).

The adsorption energy was calculated as: *E_ads_* = *E**_adsorbate/slab_* – [*E_slab_* + *E_adsorbate_*], where *E_adsorbate_*_/slab_, *E_adsorbate_* and *E_slab_* are the total energies of the species adsorbed on the slab, the isolated adsorbate, and the optimized slab, respectively. Therefore, more negative *E_ads_* values indicate stronger adsorption. The reaction energy (Δ*E*_r_) and energy barrier (*E*_a_) were calculated based on Δ*E_r_* = *E*_FS_ – *E*_IS_ and *E*_a_ = *E*_TS_ – *E*_IS_, where *E*_FS_, *E*_IS_, and *E*_TS_ were the corresponding energies of the final state (FS), initial state (IS) and transition state (TS), respectively.

***Models:*** In this calculation, the surface models of Co(111)/NC and 4Y_2_O_3_-Co(111)/NC were used to investigate the mechanism of NH_3_ decomposition. A vacuum layer of 15 A was added to minimize periodic interactions along directions perpendicular to the surface. For Co(111)/NC the surface has 48 Co, 24 C and 8 N atoms, and the bottom atoms are fixed. For 4Y_2_O_3_-Co(111)/NC, the surface has 64 Co, 8 Y, 48 C ,12 O and 18 N atoms, and the bottom atoms are fixed.

**3. Supplemental figures**


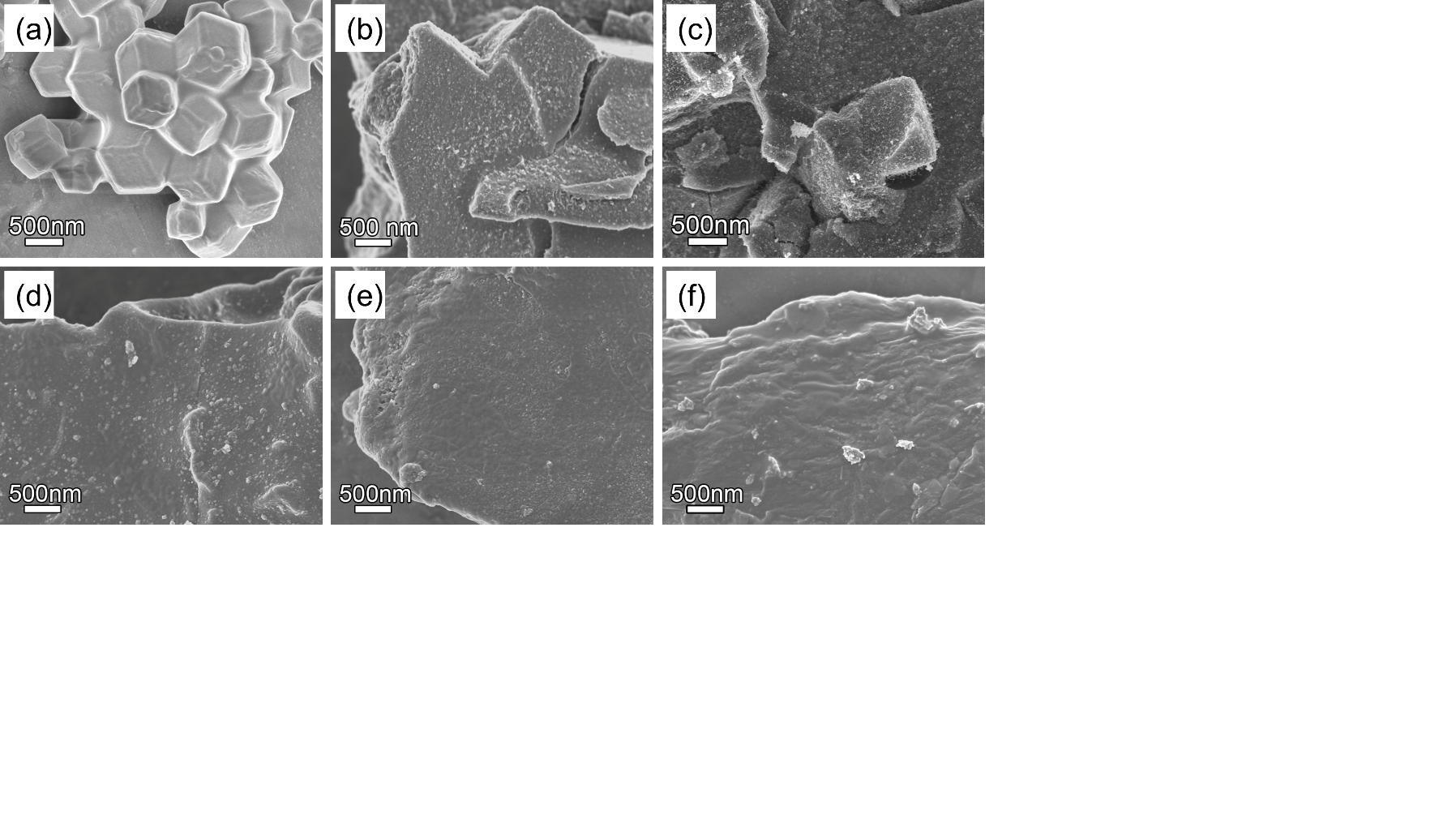


**Figure** S1 SEM images of the Y(NO_3_)_3_/ZIF-67 (a), Co/NC (b), 2Y_2_O_3_-Co/NC (c), 4Y_2_O_3_-Co/NC (d), 6Y_2_O_3_-Co/NC (e) and 8Y_2_O_3_-Co/NC (f).


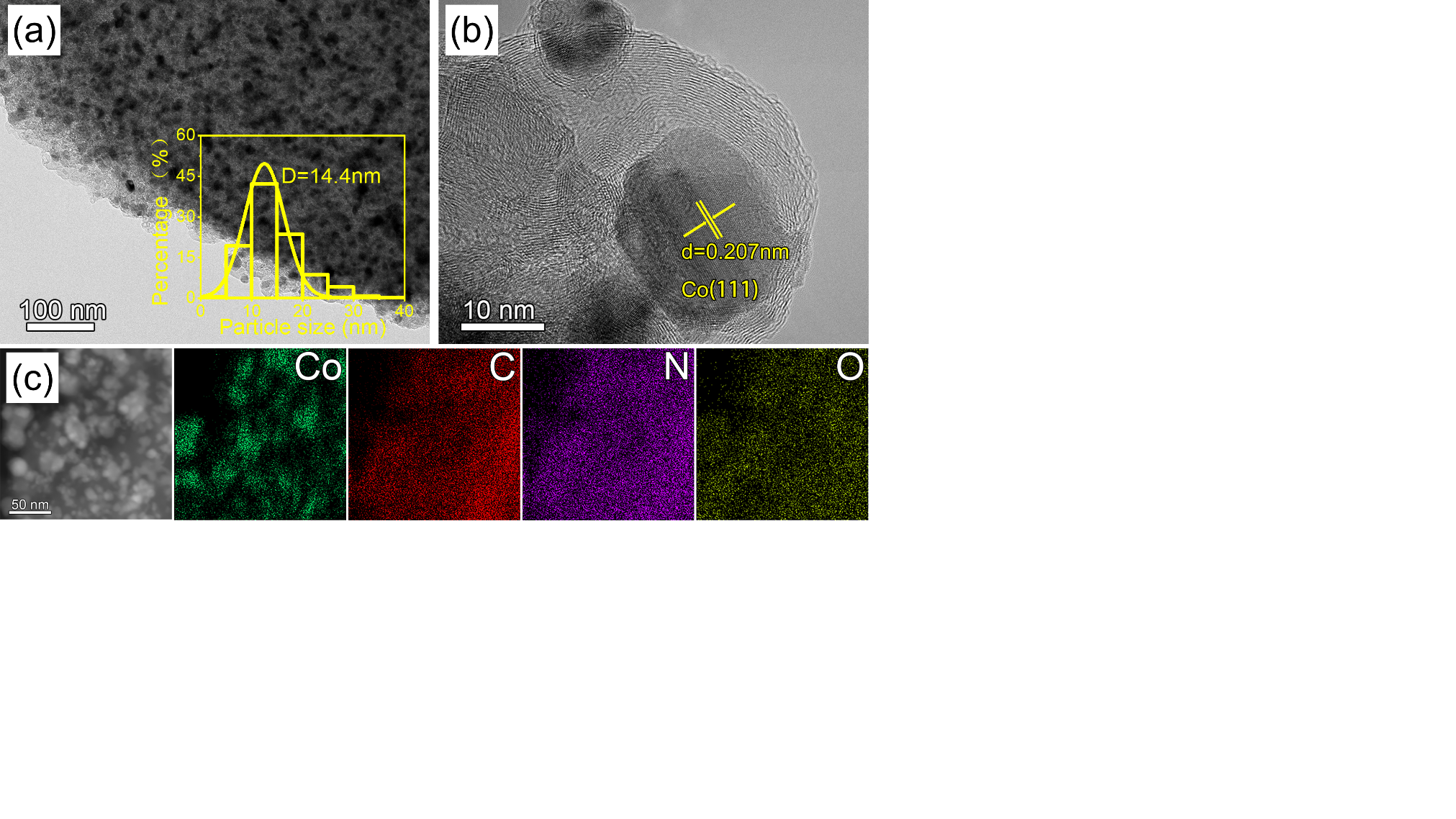


**Figure** S2 TEM image and particle size distribution profile (a). HR-TEM (b) and the corresponding elemental mapping analysis images (c) of the Co/NC catalyst.


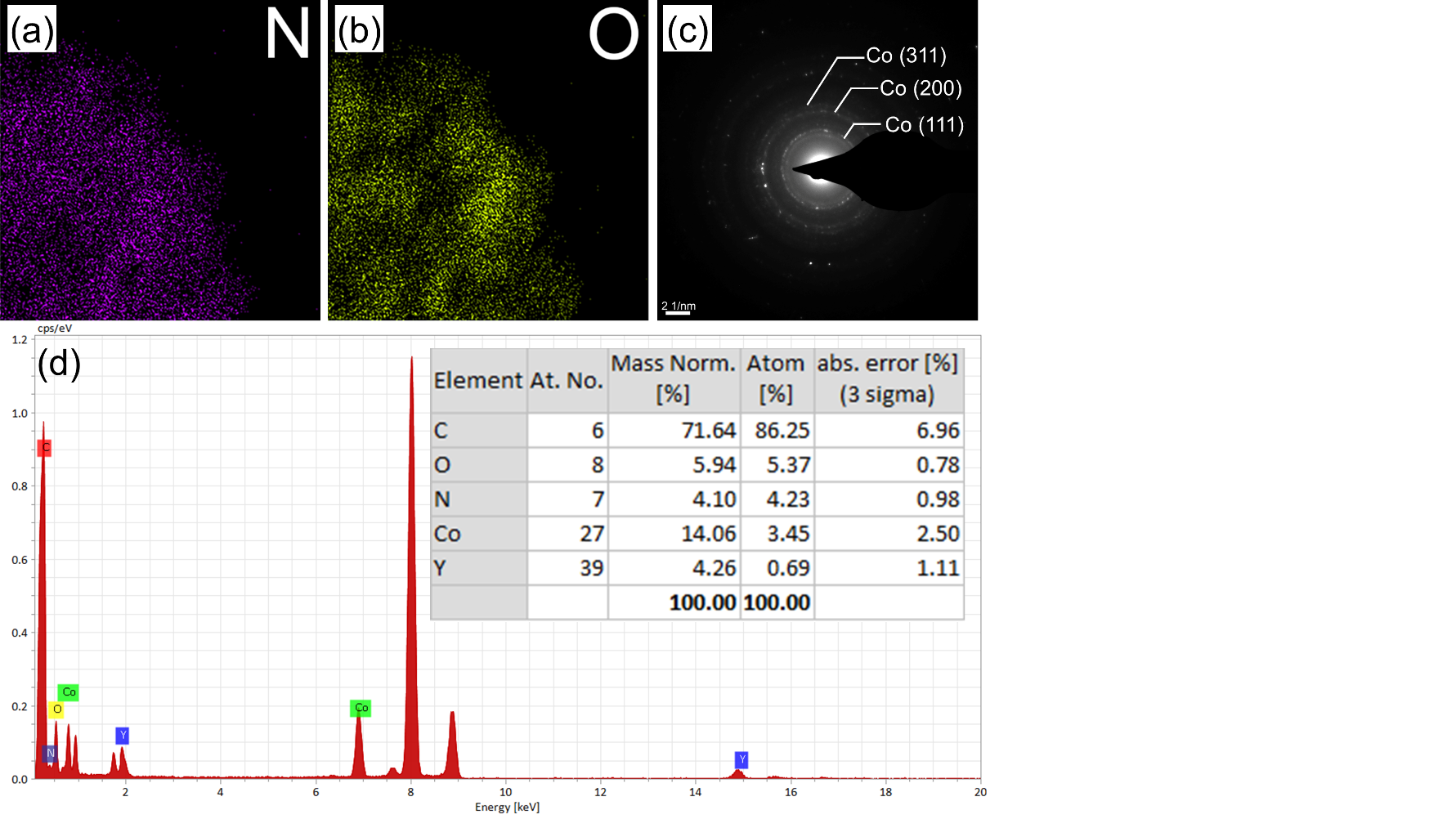


**Figure** S3 N Elemental mapping (a), O Elemental mapping (b), SAED pattern (c) and EDX spectrum (d) of the 4Y_2_O_3_-Co/NC catalyst.


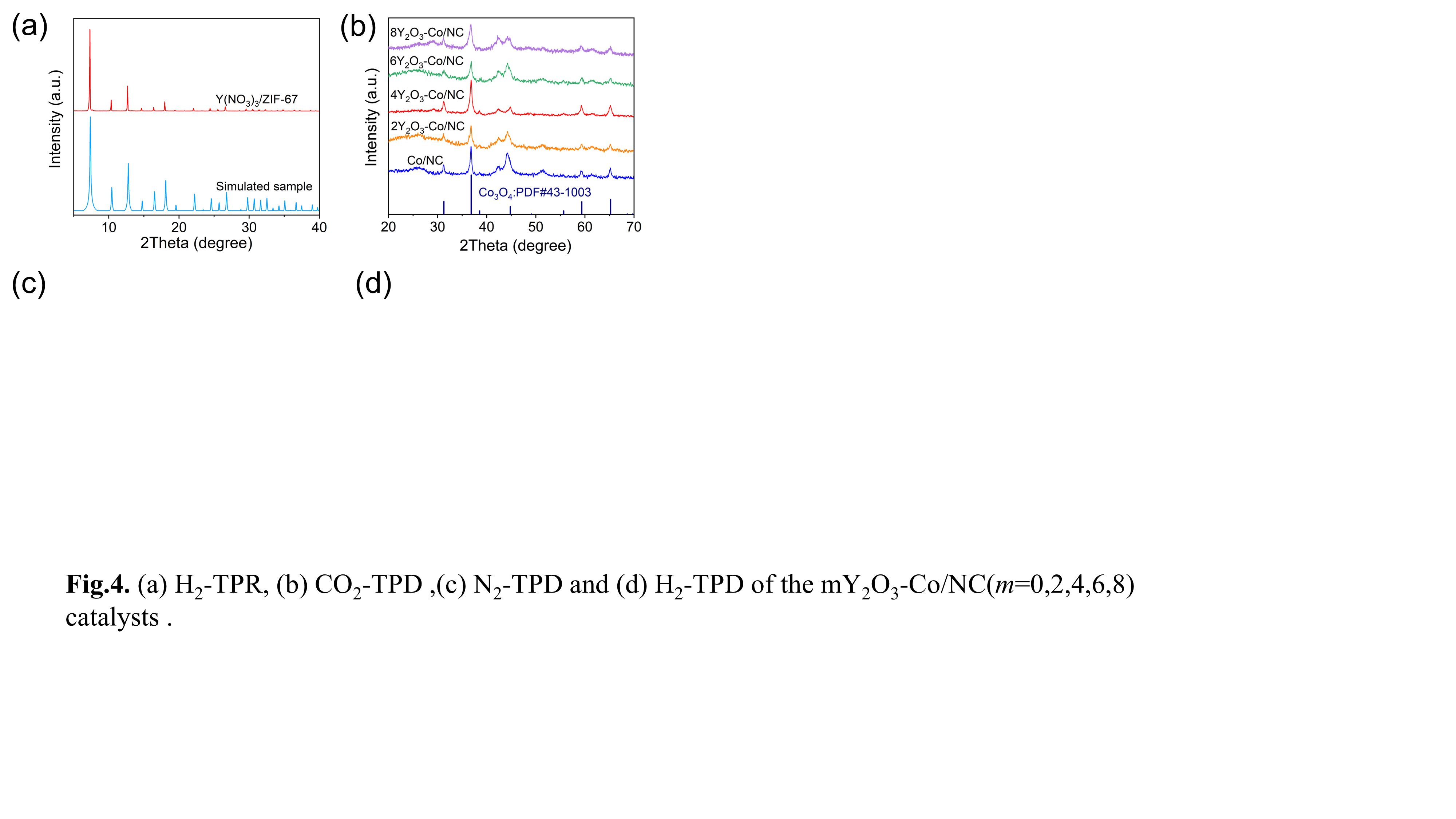


**Figure** S4 (a) XRD patterns of the Y(NO_3_)_3_/ZIF-67. (b) XRD spectrum of prepared catalysts before reduction with NH_3_.


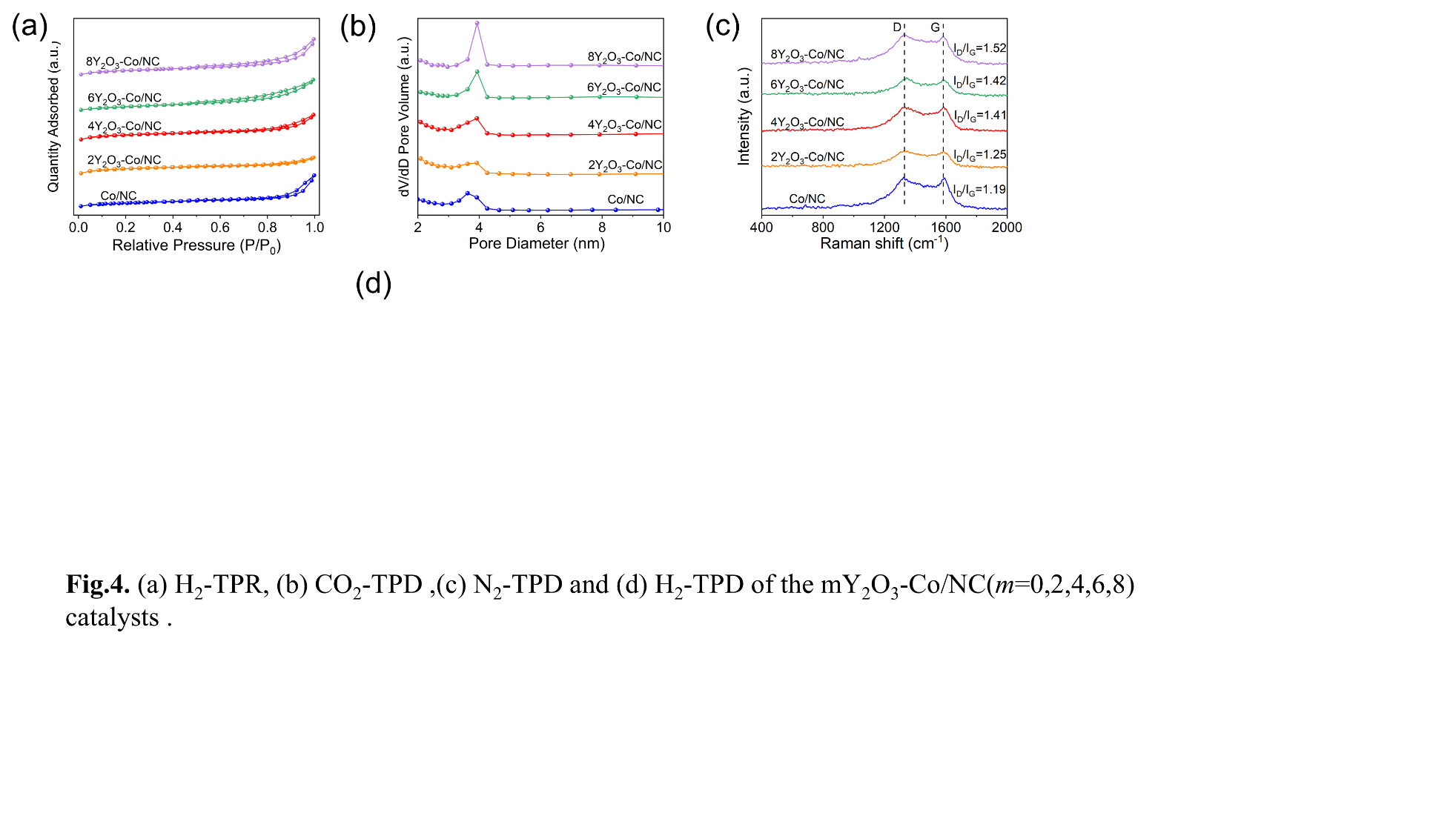


**Figure** S5 Nitrogen adsorption-desorption isotherms (a), pore size distribution curves (b) and Raman spectra (c) of prepared catalysts.


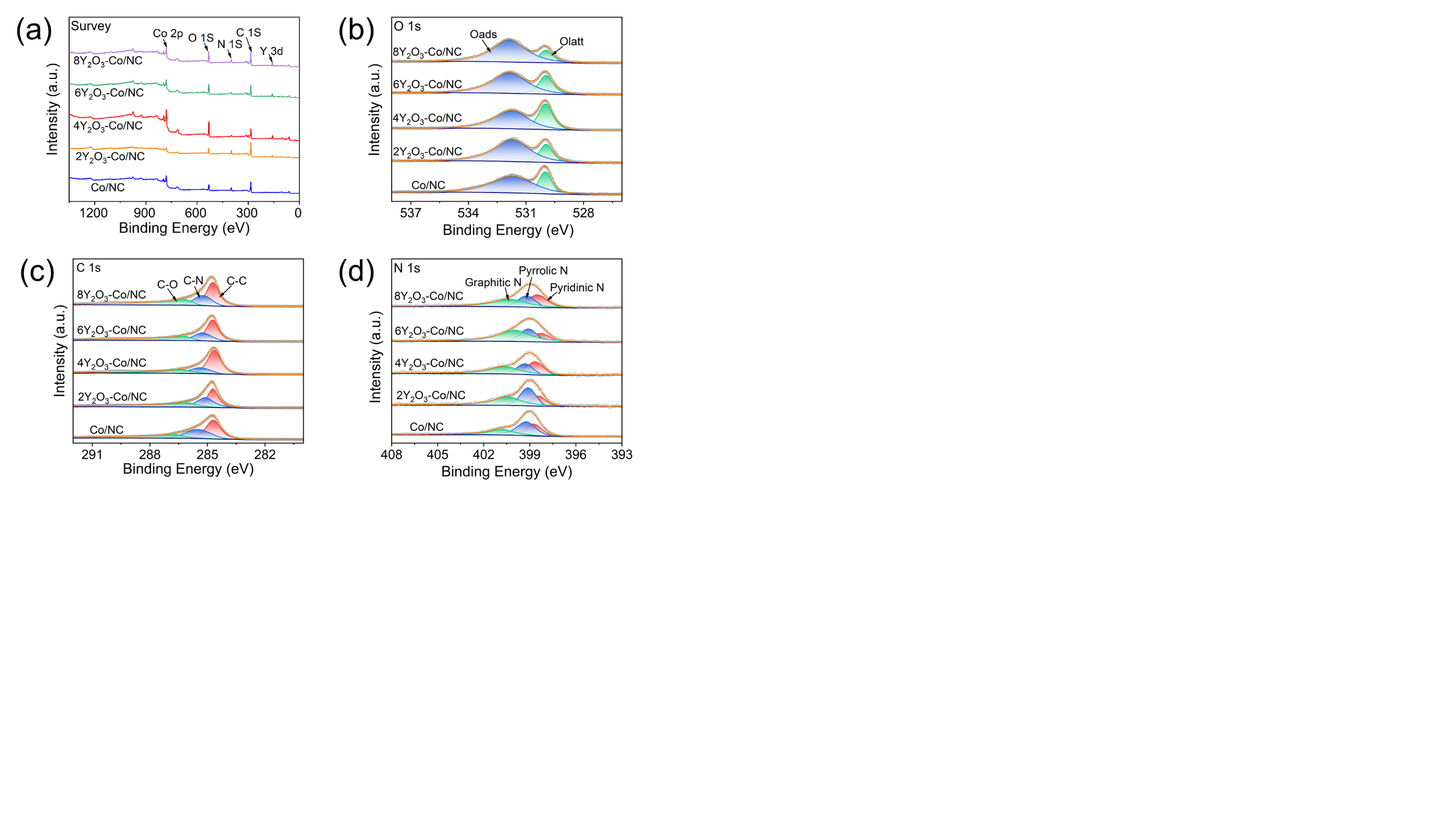


**Figure** S6 (a) S7 XPS survey spectra of prepared catalysts. High-resolution XPS spectra and the deconvolution results of prepared catalysts for O 1s (b), C 1s (c), and N 1s (d).


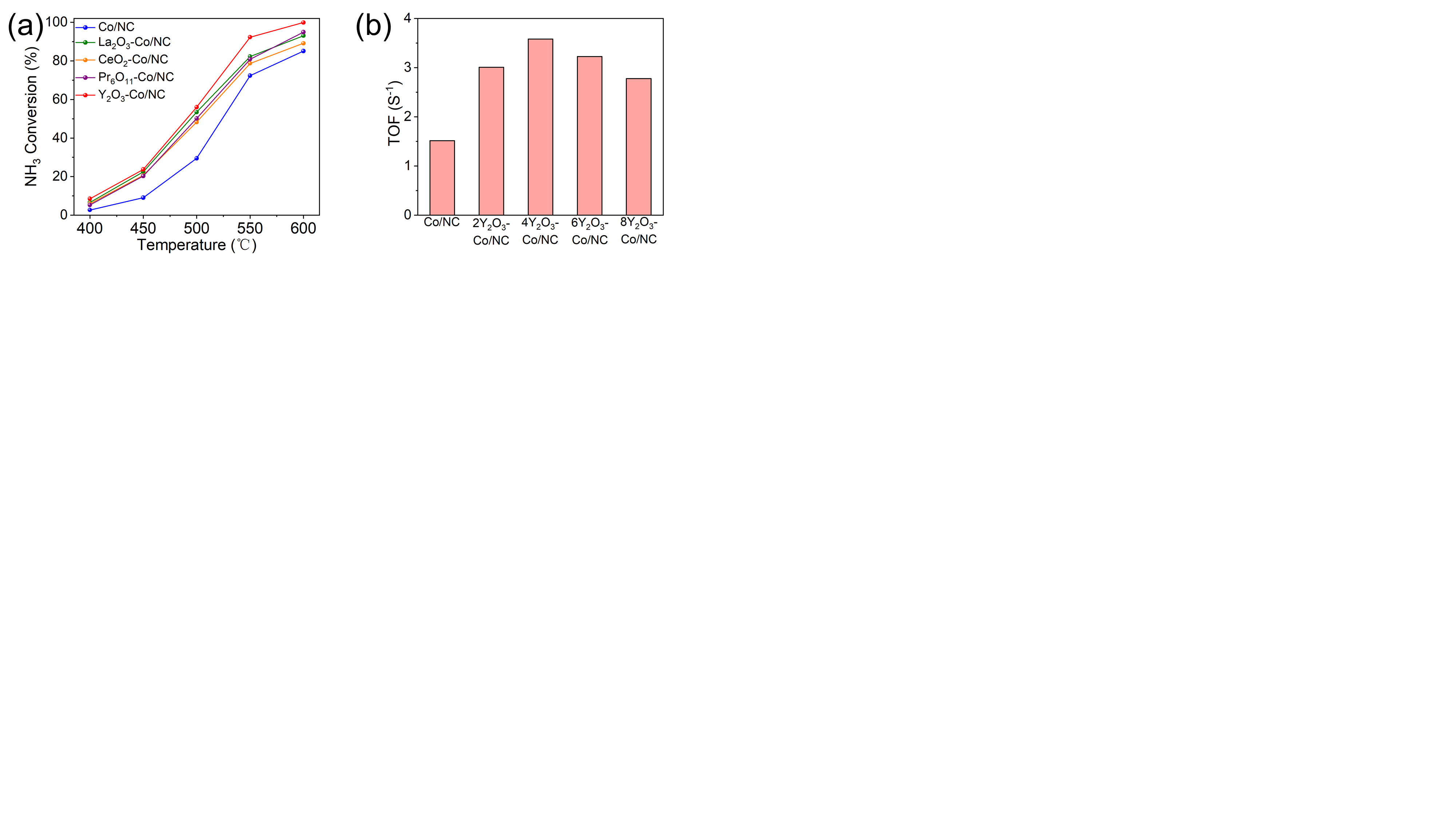


**Figure** S7 (a) Ammonia conversion as a function of temperature at a GHSV of 20,000 cm^3^·$\text{g}_{\text{cat}}^{\text{-1}}$·h^-1^. (b) TOF of prepared catalysts.


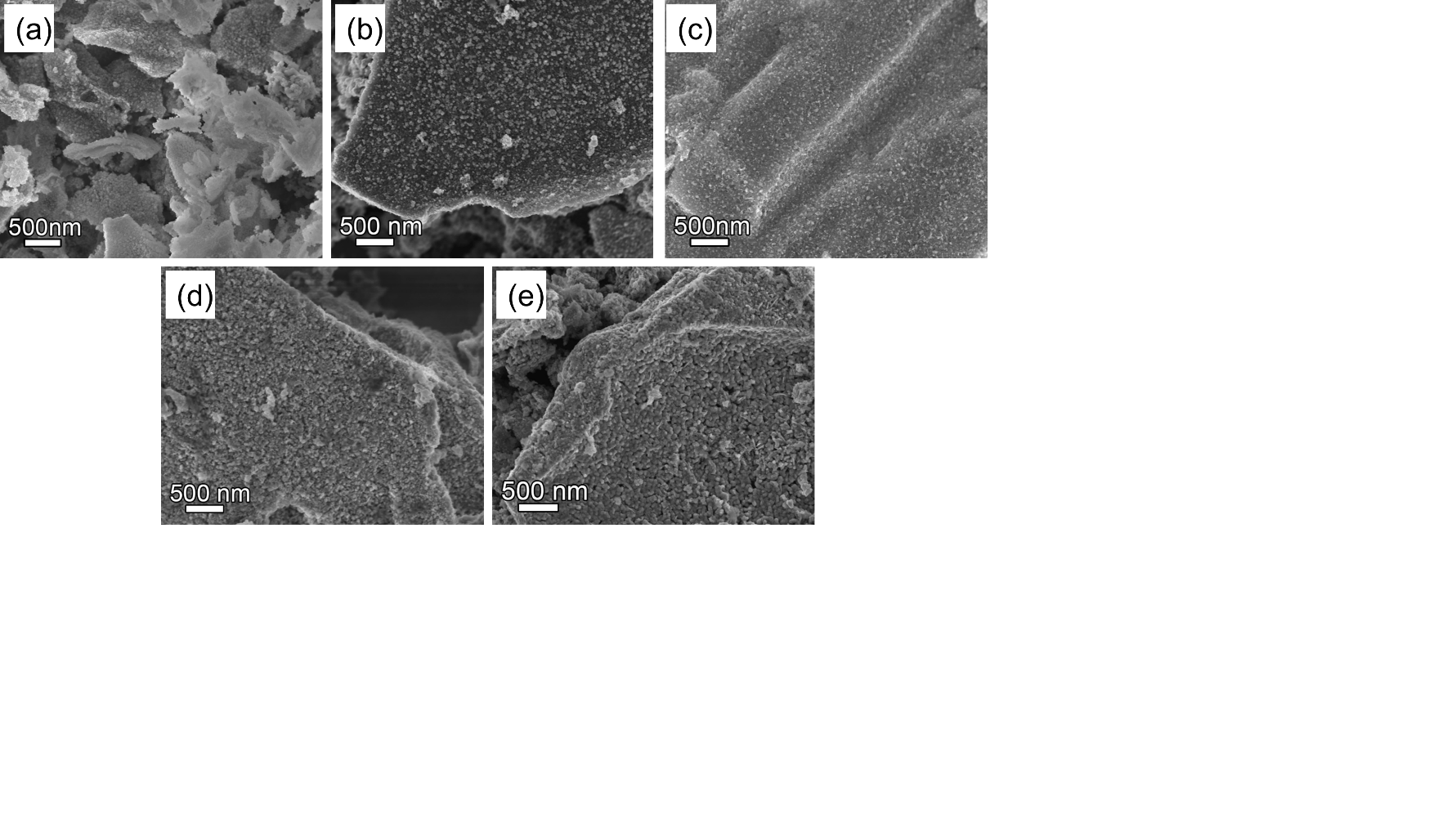


**Figure** S8 SEM images of Co/NC (a), 2Y_2_O_3_-Co/NC (b), 4Y_2_O_3_-Co/NC (c), 6Y_2_O_3_-Co/NC (d) and 8Y_2_O_3_-Co/NC catalyst (e) after stability test at 550 ℃ at a GHSV of 20,000 cm^3^·$\text{g}_{\text{cat}}^{\text{-1}}$·h^-1^ for 72h.


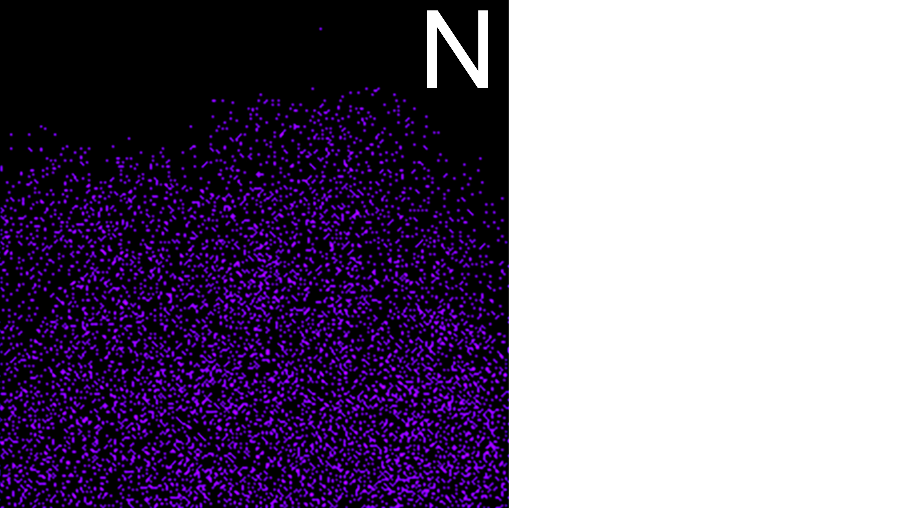


**Figure** S9 N elemental mappings of the used Co/NC catalyst.


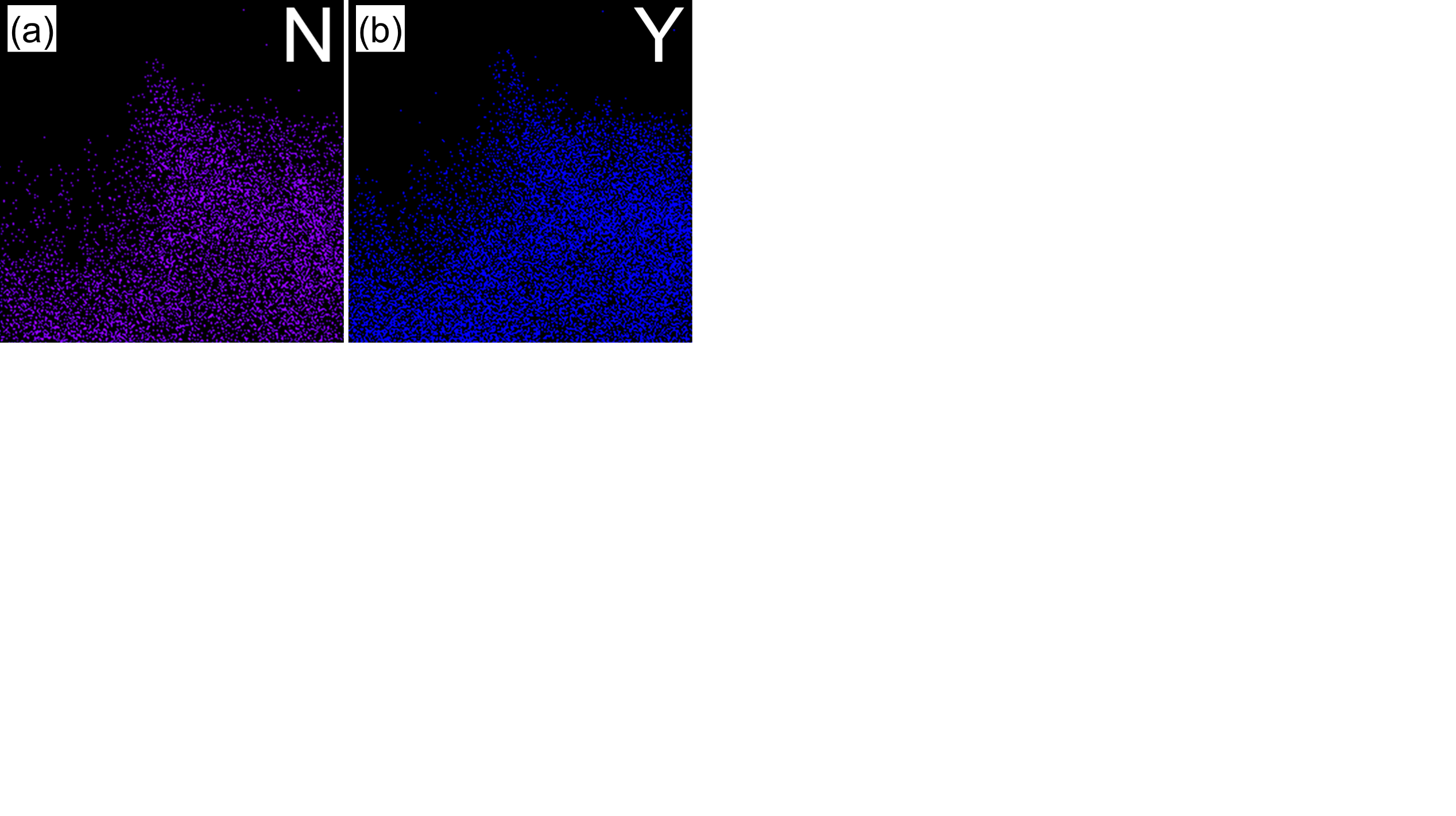


**Figure** S10 N elemental (a) and Y elemental (b) mappings of the used 4Y_2_O_3_-Co/NC catalyst.


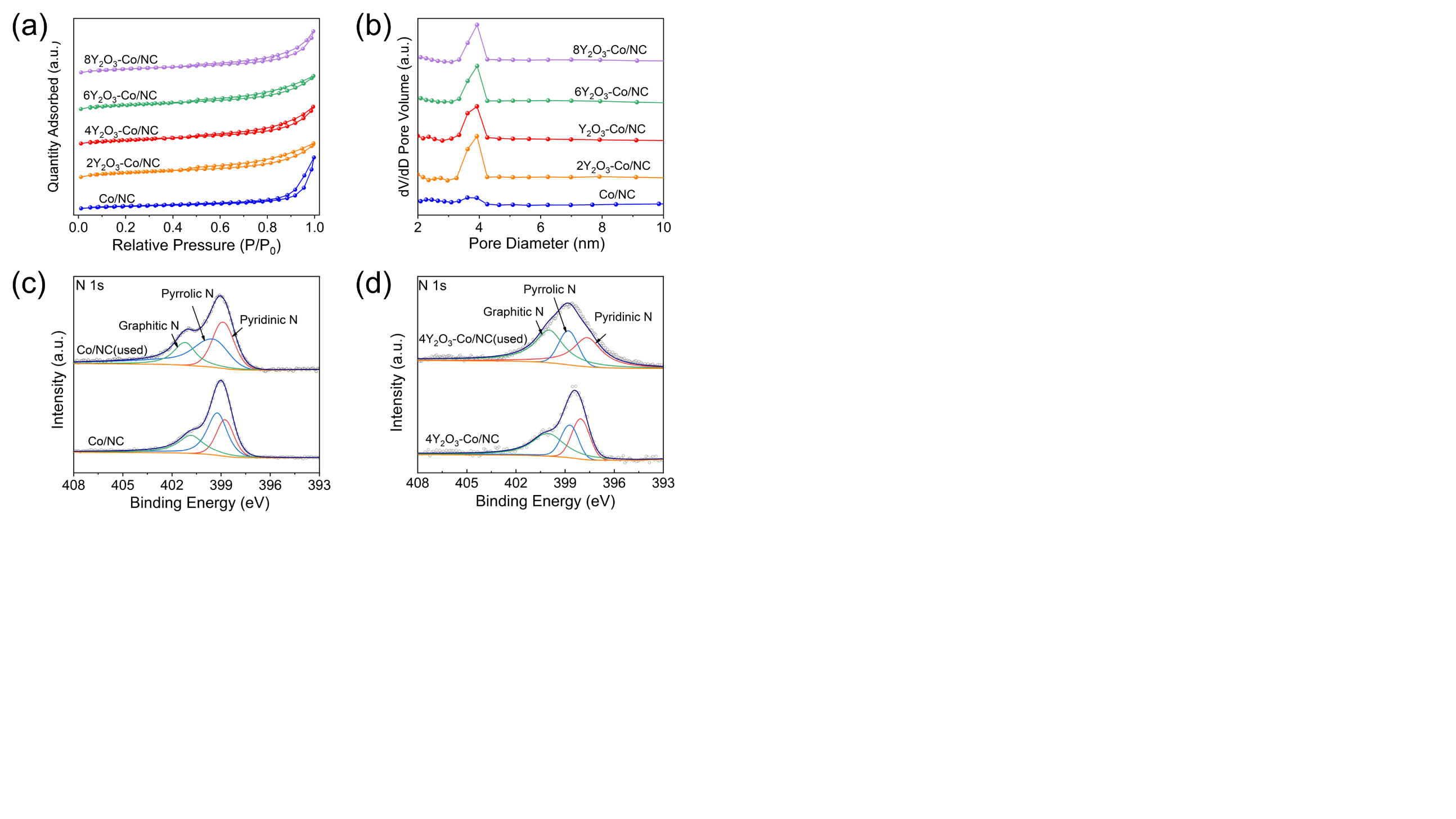


**Figure** S11 Nitrogen adsorption-desorption isotherms (a) and pore size distribution curves (b) of the used catalyst. (c) XPS N 1p spectra of the fresh and used Co/NC catalyst. (d) XPS N 1s spectra of the fresh and used 4Y_2_O_3_-Co/NC catalyst.


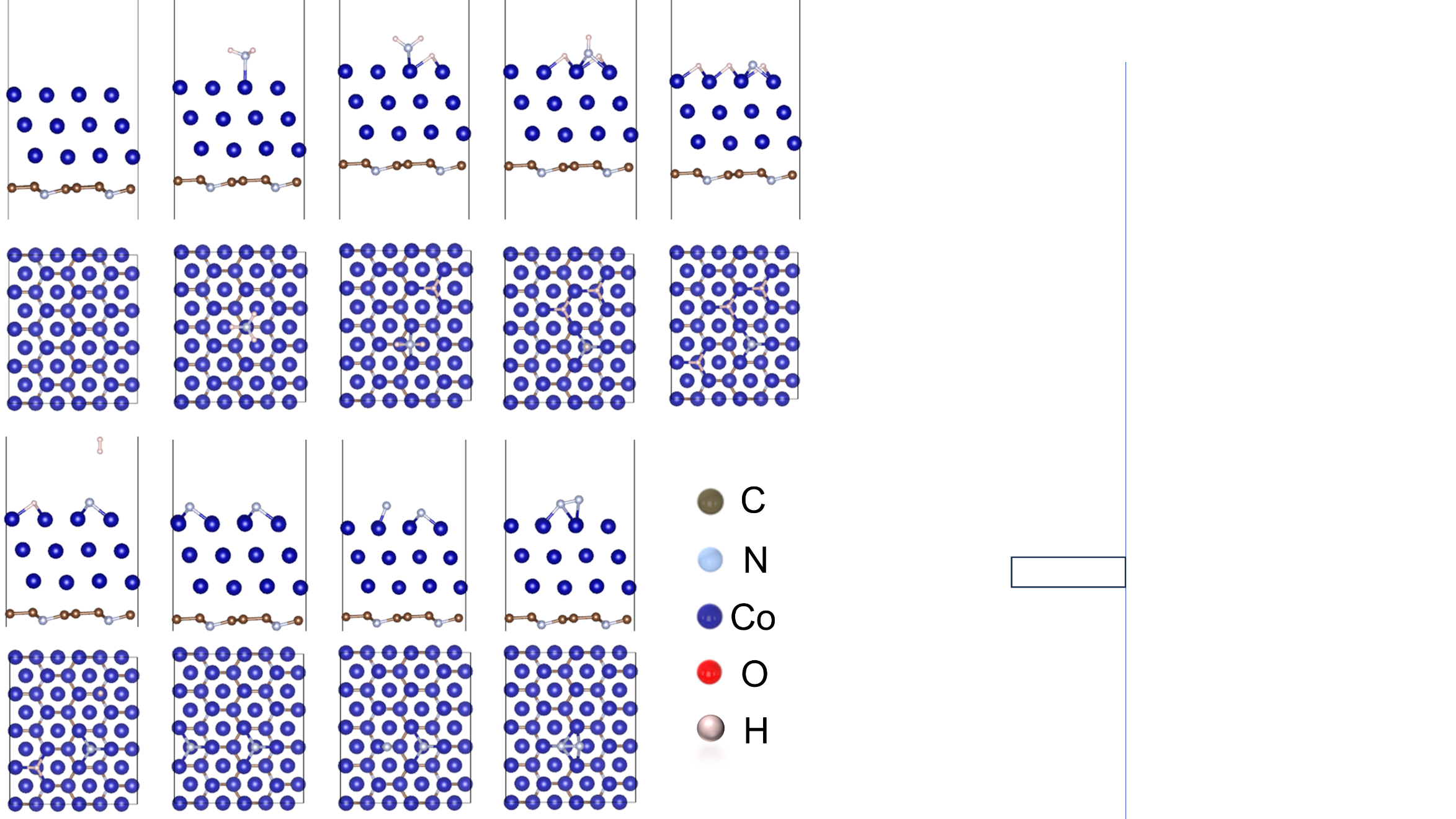


**Figure** S12 Atomic structure model of the Co/NC catalyst and the adsorbed species in the catalytic process.


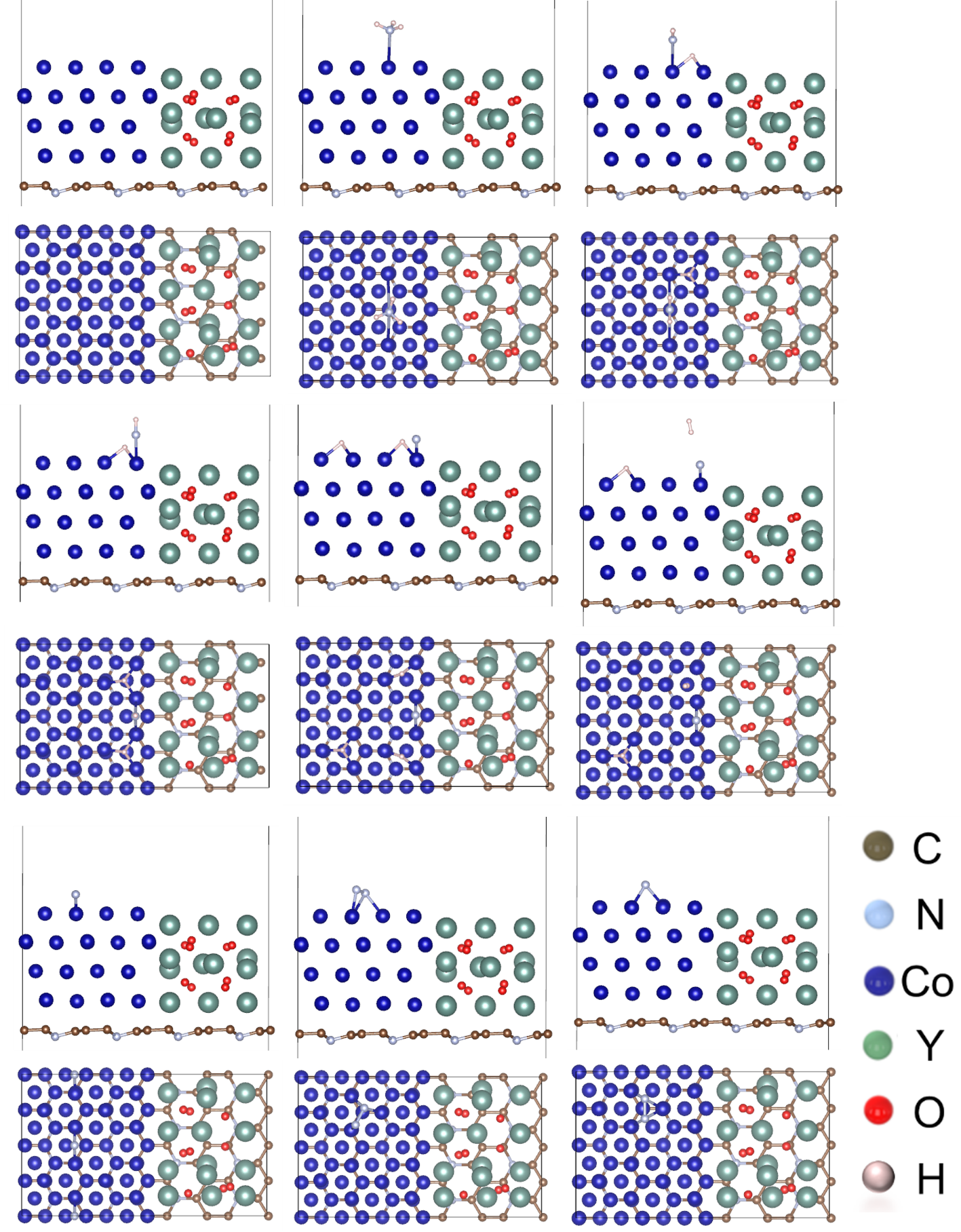


**Figure** S13 Atomic structure model of the 4Y_2_O_3_-Co/NC catalyst and the adsorbed species in the catalytic process.


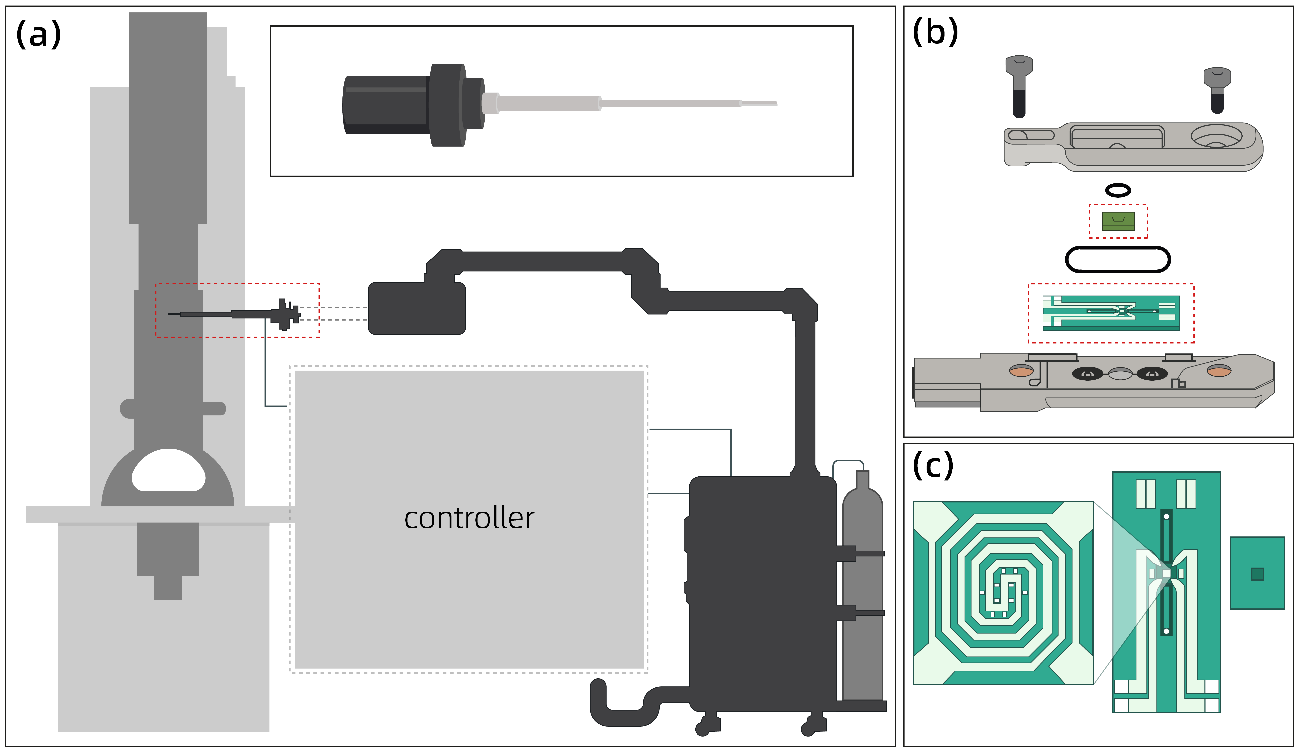


**Figure** S14 Operando TEM experimental device and chip structure schematic diagram: (a) Schematic diagram of the operando TEM and the gas supply system, with the illustration showing a magnification of the holder placed in the operando TEM. (b) Structure schematic diagram of the gas phase cabinet. (c) structure schematic diagram of the chip, magnified part is the observation area of the operando TEM.


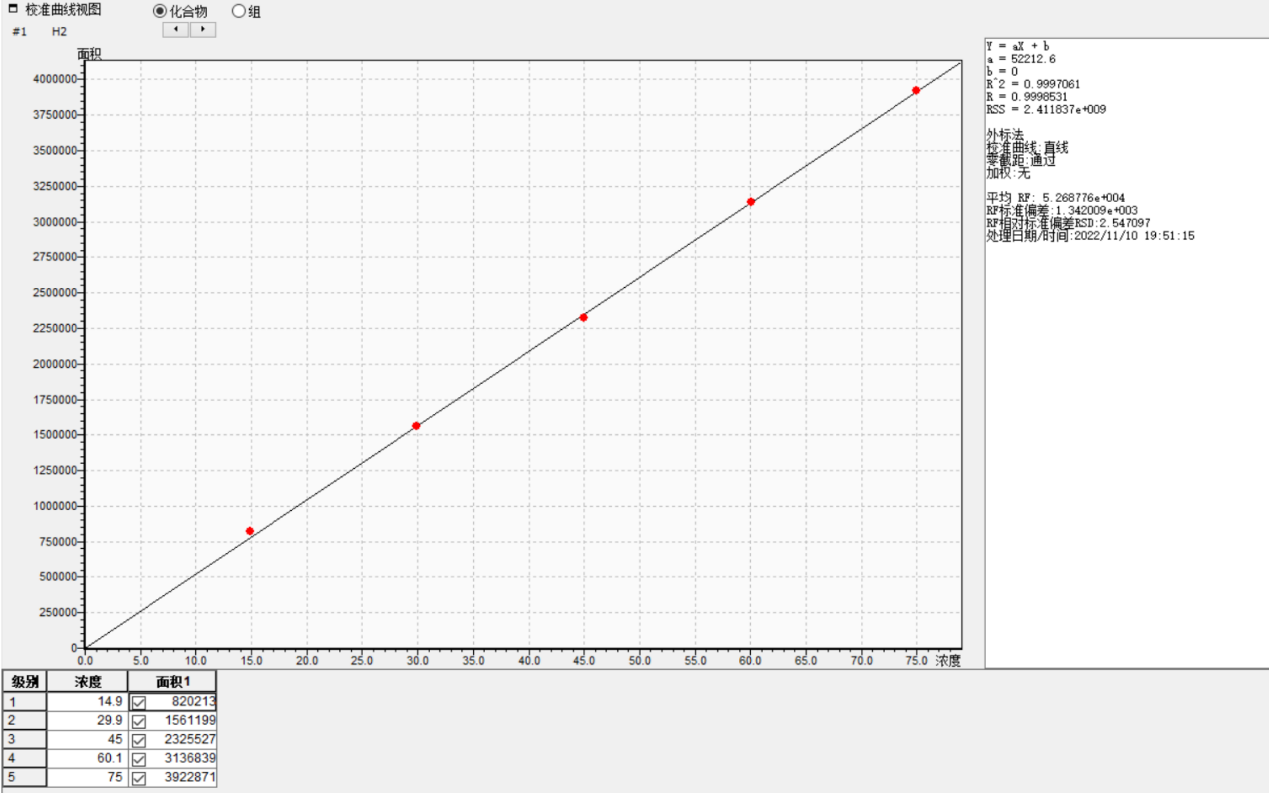


**Figure** S15 calibration curve determined by standard gases with different concentrations.

**4. Supplemental tables**

**Table** S1. The BET specific surface area, adsorption pore volume and adsorption average pore diameter of prepared catalysts before and after stability test.

| **Catalyst** | **S_bet_ (m^2^/g)** | **V_p_ (cm^3^/g^-1^)** | **D_p_ (nm)** |
| --- | --- | --- | --- |
| Co/NC | 213.4 | 0.19 | 3.7 |
| Co/NC (used) | 83.0 | 0.24 | 11.4 |
| 2Y_2_O_3_-Co/NC | 265.1 | 0.15 | 2.3 |
| 2Y_2_O_3_-Co/NC (used) | 152.5 | 0.18 | 4.8 |
| 4Y_2_O_3_-Co/NC | 237.1 | 0.18 | 3.0 |
| 4Y_2_O_3_-Co/NC (used) | 102.6 | 0.18 | 7.0 |
| 6Y_2_O_3_-Co/NC | 146.1 | 0.16 | 4.5 |
| 6Y_2_O_3_-Co/NC (used) | 93.1 | 0.15 | 6.4 |
| 8Y_2_O_3_-Co/NC | 126.1 | 0.18 | 5.7 |
| 8Y_2_O_3_-Co/NC (used) | 92.9 | 0.20 | 8.4 |

Table S2. Deconvoluted components parameters obtained in the Co 2p_1/2_ and Co 2p_3/2_ core level from the XPS spectra of prepared catalysts, used Co/NC sample and used 4Y_2_O_3_- Co/NC sample

| **Component** | Co 2p_1/2_ | | |  | Co 2p_3/2_ | | |
| --- | --- | --- | --- | --- | --- | --- | --- |
|  | Co^0^ | Co^2+^ | Co^3+^ |  | Co^0^ | Co^2+^ | Co^3+^ |
| Co/NC | 16.7 | 38.2 | 45.1 |  | 33.4 | 26.3 | 40.3 |
| 2Y_2_O_3_-Co/NC | 23.7 | 31.5 | 44.8 |  | 33.7 | 33.2 | 33.1 |
| 4Y_2_O_3_-Co/NC | 20.5 | 32.0 | 47.5 |  | 33.7 | 37.7 | 28.6 |
| 6Y_2_O_3_-Co/NC | 21.6 | 46.2 | 32.2 |  | 34.4 | 39.5 | 26.1 |
| 8Y_2_O_3_-Co/NC | 22.9 | 37.0 | 40.1 |  | 33.0 | 40.8 | 26.2 |
| Co/NC(used) | 18.3 | 45.6 | 32.1 |  | 33.0 | 27.8 | 39.2 |
| 4Y_2_O_3_-Co/NC(used) | 20.4 | 39.1 | 40.5 |  | 33.2 | 35.9 | 30.9 |

**Table** S3. Metal dispersion, specific surface area, and average particle size of Co nanoparticles of prepared catalysts determined by CO pulse chemisorption.

| **Catalyst** | **Metal dispersion**  **(%)** | **Metal surface**  **Area**  **(m^2^·g^-1^)** | **Average**  **particle diameter (nm)** |
| --- | --- | --- | --- |
| Co/NC | 2.22 | 15 | 26.9 |
| 2Y_2_O_3_-Co/NC | 2.36 | 16 | 25.2 |
| 4Y_2_O_3_-Co/NC | 2.47 | 16.7 | 24.1 |
| 6Y_2_O_3_-Co/NC | 2.36 | 15.9 | 25.2 |
| 8Y_2_O_3_-Co/NC | 2.32 | 15.7 | 25.7 |

**Table** S4. The content of Co and elements of prepared catalysts determined by ICP-OES.

| **Catalyst** | **Co (wt.%)** | **Y (wt.%)** |
| --- | --- | --- |
| Co/NC | 30.78 | — |
| 2Y_2_O_3_-Co/NC | 30.40 | 2.17 |
| 4Y_2_O_3_-Co/NC | 29.89 | 4.29 |
| 6Y_2_O_3_-Co/NC | 29.40 | 6.19 |
| 8Y_2_O_3_-Co/NC | 29.03 | 8.32 |

**Table** S5. Comparison of the NH_3_ decomposition reaction activity of prepared catalysts with the recently reported results.

| **Catalyst** | **Temp.**  **(℃)** | **GHSV**  **(cm^3^·**$\text{g}_{\text{cat}}^{\text{-1}}$  **·h^-1^)** | **Conv.**  **(%)** | **H_2_ form. rate**  **(mmol·**  $\text{g}_{\text{cat}}^{\text{-1}}$**·min^-1^)** | **Ref.** |
| --- | --- | --- | --- | --- | --- |
| Co/CeO_2_-3DOM | 600 | 6,000 | 62 | 6.7 | [S1] |
| Cs-Co_3_Mo_3_N | 550 | 6,000 | 100 | 4.5 | [S2] |
| Fe/Co/NiMo_3_N | 600 | 6,000 | 100 | 4.5 | [S3] |
| Co_3_Mo_3_N | 550 | 6,000 | 80 | 5.3 | [S4] |
| CoMoN_2_/CNT | 600 | 11,000 | 71.5 | 8.8 | [S5] |
| CoNi/Zr-Y_2_O_3_ | 550 | 9,000 | 90.9 | 9.1 | [S6] |
| 20Co-10Ni/Y_2_O_3_ | 550 | 9,000 | 85 | 8.5 | [S7] |
| 90CoAl | 550 | 18,000 | 90 | 18.1 | [S8] |
| Fe-Al_2_O_3_ | 550 | 15,000 | 62 | 10.5 | [S9] |
| Fe/SiO_2_ | 600 | 15,000 | 86 | 14.5 | [S10] |
| Fe-Mg | 550 | 6,000 | 86 | 5.8 | [S11] |
| CoO_x_@C | 500 | 15,000 | 55 | 9.3 | [S12] |
| Co-Ax-21 | 500 | 5,200 | 60 | 3.5 | [S13] |
| Co-NC | 500 | 30,000 | 80 | 27 | [S14] |
| LaCoO_x_/Co@NC/SBA-15 | 550 | 30,000 | 100 | 33.8 | [S15] |
| 20Co/La-MgO(5) | 500 | 22,000 | 91 | 22.6 | [S16] |
| 4Y_2_O_3_-Co/NC | 550 | 20,000 | 92.3 | 20.6 | This work |

**Table** S6. Adsorption energies (*E*_ads_, eV) and reaction energies (Δ*E*_r_, eV) for the pathways of NH_3_ decomposition on the Co(111)/NC and 4Y_2_O_3_-Co(111)/NC surfaces.

| **Reaction** | **Co(111)/NC** | |  | **4Y_2_O_3_-Co(111)/NC** | |
| --- | --- | --- | --- | --- | --- |
|  | ***E*_ads_** | **Δ*E*_r_** |  | ***E*_ads_** | **Δ*E*_r_** |
| NH_3_^*^ +* → NH_2_^*^ + H^*^ | -0.81 | -0.05 |  | -0.81 | -0.33 |
| NH_2_^*^ → NH^*^ + H^*^ | -7.62 | -0.34 |  | -7.89 | -0.07 |
| NH^*^ → N^*^ + H^*^ | -14.43 | 0.21 |  | -14.43 | -0.44 |
| H^*^ + H^*^ → H_2_ + 2* | -20.47 | 1.13 |  | -21.12 | 0.76 |

**Table** S7. Adsorption energies (*E*_ads_, eV), energy barriers (*E*_a_, eV) and reaction energies (Δ*E*_r_, eV) for the pathway of desorption of N_2_ on the Co(111)/NC and 4Y_2_O_3_-Co(111)/NC surfaces.

| **Catalyst** | **N^*^ + N^*^ →N_2_ + 2*** | | |
| --- | --- | --- | --- |
|  | ***E*_ads_** | ***E*_a_** | **Δ*E*_r_** |
| Co(111)/NC | -17.81 | 2.51 | 1.19 |
| 4Y_2_O_3_-Co(111)/NC | -18.81 | 2.18 | 1.14 |

**References**

1. A. Srifa, K. Okura, T. Okanishi, H. Muroyama, T. Matsui and K. Eguchi, *Applied Catalysis B-Environmental*, **2017**, 218, 1-8.
2. C. Q. Huang, Y. Z. Yu, X. Y. Tang, Z. Y. Liu, J. Zhang, C. Z. Ye, Y. Ye and R. B. Zhang, *Applied Surface Science*, **2020**, 532.
3. A. Srifa, K. Okura, T. Okanishi, H. Muroyama, T. Matsui and K. Eguchi, *Catalysis Science & Technology*, **2016**, 6, 7495-7504.
4. L. A. Jolaoso, S. F. Zaman, S. Podila, H. Driss, A. A. Al-Zahrani, M. A. Daous and L. Petrov, *International Journal of Hydrogen Energy*, **2018**, 43, 4839-4844.
5. Z. H. Zhao, H. B. Zou and W. M. Lin, *Journal of Rare Earths*, **2013**, 31, 247-250.
6. C. J. Li, L. X. Guo, G. H. Chen, Y. Q. Fu, X. X. Zhang, Y. Q. Zou, J. H. Duan and W. W. Wang, *Colloids and Surfaces a-Physicochemical and Engineering Aspects*, **2023**, 671.
7. H. H. Li, L. X. Guo, J. N. Qu, X. X. Fang, Y. Q. Fu, J. H. Duan, W. W. Wang and C. J. Li, *International Journal of Hydrogen Energy*, **2023**, 48, 8985-8996.
8. Z. S. Zhang, X. P. Fu, W. W. Wang, Z. Jin, Q. S. Song and C. J. Jia, *Science China-Chemistry*, **2018**, 61, 1389-1398.
9. J. S. Valente, J. Hernandez-Cortez, M. S. Cantu, G. Ferrat and E. López-Salinas, *Catalysis Today*, **2010**, 150, 340-345.
10. M. Feyen, C. Weidenthaler, R. Güttel, K. Schlichte, U. Holle, A. H. Lu and F. Schüth, *Chemistry-a European Journal*, **2011**, 17, 598-605.
11. S. Podila, H. Driss, S. F. Zaman, A. M. Ali, A. A. Al-Zahrani, M. A. Daous and L. A. Petrov, *International Journal of Hydrogen Energy*, **2020**, 45, 873-890.
12. L. Li, R. Y. Jiang, W. Chu, H. Cang, H. W. Chen and J. L. Yan, *Catalysis Science & Technology*, 2017, **7**, 1363-1371.
13. L. Wang, Y. H. Yi, Y. Zhao, R. Zhang, J. L. Zhang and H. C. Guo, *ACS Catalysis*, **2015**, 5, 4167-4174.
14. G. R. Li, H. F. Zhang, X. T. Yu, Z. P. Lei, F. X. Yin and X. B. He, *International Journal of Hydrogen Energy*, **2022**, 47, 12882-12892.
15. X. Han, M. H. Hu, J. C. Yu, X. Xu, P. Jing, B. C. Liu, R. Gao and J. Zhang, *Applied Catalysis B-Environmental*, 2023, **328**, 122534.
16. X. C. Hu, W. W. Wang, Z. Jin, X. Wang, R. Si and C. J. Jia, *Journal of Energy Chemistry*, **2019**, 38, 41-49.
